# Supplementary material for: Exploration of forestry carbon sequestration practice path in Guizhou province-based on evolutionary game model
Source: PLoS One. 2024 Dec 13;19(12):e0314805. doi: 10.1371/journal.pone.0314805 (PMC11643302; doi:10.1371/journal.pone.0314805)
Supplement: S1 File — I have downloaded the complete yearbook report and uploaded it as an attachment for data support. (DOCX) [file pone.0314805.s001.docx]

Forestry Yearbook of Guizhou Province in 2023

[Overview] In 2023, the Forestry Bureau of Guizhou Province thoroughly implemented 's socialist ideology with Chinese characteristics in the new era and the spirit of General Secretary 's important speech on inspecting Guizhou, firmly adhered to the two bottom lines of development and ecology, coordinated the systematic management of mountains, rivers, forests, fields, lakes, grass and sand, adhered to the overall situation of high-quality development, and successfully completed the annual objectives and tasks. The forest area of the province is 11.2667 million hectares, the forest area is 11.0667 million hectares, and the forest coverage rate is 63%. The grassland area is 189300 hectares and the wetland area is 256700 hectares. After integration and optimization, there are 237 nature reserves in the province, covering an area of 1.7705 million hectares. There are 8 world natural heritage sites, 11 national nature reserves, 18 national scenic spots, 8 national geological parks, 35 national wetland parks and 24 national forest parks in the province. The total output value of forestry industry reached 424.7 billion yuan, of which the output value of under-forest economy reached 75.9 billion yuan, and the output value of characteristic forestry industry reached 29.5 billion yuan.

[Land greening] Adhering to the principle of "forest is suitable for forest and grass is suitable for grass", a total of 214,000 hectares of afforestation have been completed throughout the year, and the task of afforestation has been accurately mapped. 134000 hectares of low-yielding forests have been transformed, 50000 hectares of tree species have been adjusted, and 122600 hectares of new national reserve forest projects have been built. It has completed 314600 hectares of precise forest quality improvement projects and 744 square kilometers of comprehensive control of rocky desertification. The comprehensive vegetation coverage of grassland reached 89.6%, and 7880 hectares of grass planting and improvement were completed. To carry out rural greening and beautification, 800 hectares of rural greening and beautification were completed throughout the year, and the coverage rate of village greening reached 47.21%. We consolidated the achievements of returning farmland to forests and completed the replanting of 206,600 hectares.

[Protection of Forestry Resources] First, we will continue to carry out forest supervision and special actions to combat deforestation and grassland destruction. The rectification rate of forest inspection cases in Guizhou Province in 2022 is 100%, which is one of the first three provinces in the country to complete the rectification. In 2023, the number of forest-related cases, the area of illegally occupied forest land and the accumulation of illegal logging in Guizhou Province decreased by more than 50% compared with the same period last year, while the special action of combating deforestation and grassland destruction was carried out simultaneously with forest supervision, and 42497 clues were investigated. The second is to strengthen the rectification of key problems. The provincial forest chief office has listed and supervised 10 areas with prominent problems (5 cases of forest land destruction and 5 cases of forest destruction) and 15 typical cases (8 cases of forest land destruction and 7 cases of forest destruction). The cities and counties involved in the listing supervision have carried out on-site supervision at the provincial level one by one, and all the supervised areas have completed the rectification task. Thirdly, we should strengthen the system construction. Notice of the Provincial Forestry Bureau on the Guarantee of Forestry Elements for Major Projects and Key Projects at or above the Provincial Level in the First Quarter of 2023 (Qianlin Zihan [2023] 13), and Notice of the Provincial Forestry Bureau on the Implementation of the Reporting System for the Advance Use of Forest Land for Controlled Projects of Key Infrastructure Projects at or above the Provincial Level. Supervise and guide the competent forestry departments of cities and counties to do a good job in ensuring forest land elements. Together with the Guiyang Commissioner's Office of the State Forestry and Grassland Administration, the Guizhou Provincial High Court, the Provincial Procuratorate, the Provincial Public Security Department and the Provincial Department of Ecology and Environment, we formulated and promulgated the Opinions on the Subscription of Forestry Carbon Sequestration for Compensation for Damage to the Ecological Environment in Cases of Destruction of Forest Resources (Trial Implementation) to solve the problem of ecological restoration in cases of destruction of forest resources.

On May 8, 2023, Guizhou Province held a teleconference on the deployment of special actions to combat deforestation and grassland destruction, provided by the Provincial Forestry Bureau

[Wildlife Protection] The People's Government of Guizhou Province revised and issued the List of Key Protected Wild Plants in Guizhou Province and the List of Key Protected Wild Animals in Guizhou Province, and issued the Key Protected Areas of Migratory Passages in Guizhou Province (the first batch). We will continue to do a good job in the investigation, rescue and protection of the background resources of wild animals and plants, effectively protect rare and endangered species such as Guizhou golden monkey, Fanjingshan fir and Cathaya argyrophylla, and newly discover five species of national first and second-class protected wild plants such as Huotong and Baiju in Guangxi. Firmly carry out the "Qingfeng" and "Net Shield" actions, and establish a joint meeting and law enforcement coordination mechanism to combat wildlife crimes at the provincial, municipal and county levels. Build a "forestry system + social forces" wildlife shelter and rescue system with Guizhou characteristics, and promote the construction of five wildlife shelter and rescue centers (stations) such as Guiyang in an orderly manner. Establish and strengthen the monitoring system of terrestrial wildlife epidemic foci and diseases, build two national leading intelligent terrestrial wildlife epidemic foci and diseases preliminary inspection laboratories with high standards, and enhance the rapid monitoring and response capabilities of epidemic foci and diseases. Complete the survey of alien invasive species in the forest, grass and wetland ecosystem. The damage caused by wildlife should be included in forestry insurance to properly resolve the demands of the masses.

[Prevention and control of forest pests] 183,293 hectares of forest pests occurred in Guizhou Province in the whole year, including 19,007 hectares of diseases, 157,336 hectares of pests, 3,207 hectares of pikas and 3,743 hectares of harmful plants. In the occurrence area, the disaster area of forest pests is 0.059 million hectares, the disaster rate is 0.0548 ‰; the control area is 173,182 hectares, of which the pollution-free control area is 171,859 hectares, the pollution-free control rate is 99.24%; In spring, 9993.49 hectares of seedling producing areas were quarantined, with a 68782 of 91000 seedlings, and the quarantine rate of seedling declaring producing areas reached 100%. The special action of "Protecting Pine 2023" was carried out in a down-to-earth manner. A total of 1698 law enforcement vehicles and 5114 law enforcement officers were dispatched to supervise and inspect 3291 wood-related enterprises, 24 batches of imported pine were supervised and inspected, and the prevention and control of pine wood line disease was included in the nine-year compulsory education textbooks, which was the first in the country. A total of 38 cases were investigated and handled in the province, with a fine of 163800 yuan, all of which were administrative cases, with 49.50 cubic meters of infected wood and 50 packaging materials disposed of.

[Forest Fire Prevention] On March 21, the Forestry Bureau of Guizhou Province organized a joint meeting on forest and grassland fire prevention of the competent departments of forest and grassland in Guizhou, Guizhou, Sichuan, Chongqing, Tibet, Hunan and Guilin, and signed the Cooperation Agreement on Joint Prevention and Control of Forest and Grassland Fire Prevention of the competent departments of forest and grassland in Guizhou, Hunan and Guangxi Zhuang Autonomous Region. The Forestry Bureau of Guizhou Province, together with the Provincial Forest Prevention and Control Office, the Provincial Public Security Department and the Provincial Emergency Response Department, jointly issued the Notice on Organizing the Special Action to Investigate and Rectify the Hidden Dangers of Forest and Grassland Fires and Investigate and Punish the Illegal Use of Fire, and the Provincial Forestry Bureau issued the 2023 Action Plan for the Special Investigation and Rectification of the Hidden Dangers of Major Accidents in the Forestry and Grass Industry of Guizhou Province to carry out "two special actions" in the province. A total of 270 people from 9 teams of forestry systems in various cities (autonomous prefectures) were organized to participate in the competition of fire prevention skills. To formulate and issue the Work Program for the Investigation of Forest and Grassland Fire Prevention and Control in Baoxian County from 2023 to 2024, and arrange the cadres of the Provincial Forestry Bureau to continue to carry out the investigation of forest and grassland fire prevention and control in Baoxian County. Take the lead in issuing the Notice of the Provincial Forest Prevention and Control Office on the Key Work of Forest and Grassland Fire Prevention Period from 2023 to 2024. The Prompt Letter on Strengthening Forest and Grassland Fire Prevention and Control was drafted and submitted for examination, which was signed by the provincial chief forester and issued to the chief forester of each city (autonomous prefecture). The Notice of Guizhou Forestry Bureau on Comprehensively Strengthening the Work of Forest and Grassland Fire Prevention and Control in the Forestry System under the New Situation was issued. Implementing the demonstration project of intelligent forest fire monitoring and purchasing service in key forest fire prevention areas, striving for the implementation of provincial financial funds totaling 27.6 million yuan in three years, and adding 218 remote video surveillance front-end points for forest fire in key forest areas of the province.

[Forestry industry] First, the area of under-forest economic management and utilization of forest land reached 2.3113 million hectares, with 3.15 million employees. The Special Statistical Investigation System of Under-forest Economy in Guizhou Province was approved by the Provincial Statistical Bureau and included in a village-level table to carry out a special statistical investigation. Secondly, the province has completed the expansion and transformation of the industrial base of characteristic forestry (bamboo, Camellia oleifera, Zanthoxylum bungeanum, Gleditsia sinensis and Idesia polycarpa) of 98200 hectares, with a total scale of 803300 hectares (including 337300 ha of bamboo, 261600 ha of Camellia oleifera, 117000 ha of Zanthoxylum bungeanum, 67700 ha of Gleditsia sinensis and 197000 ha of Idesia polycarpa). The total scale increased by 4.5% year on year. Third, we should firmly promote the construction of woody grain and oil industry. We will expand the planting base of Camellia oleifera by 15,500 hectares and the newly built planting base of Idesia polycarpa by 19,700 hectares, and promote the provincial demonstration project of Idesia polycarpa industry by 14,600 hectares. The State Forestry and Grassland Administration issued "Yuping County of Guizhou Province to promote the upgrading of Camellia oleifera industry" and "Liping County of Guizhou Province to explore the development model of" village withdrawal management "of Camellia oleifera" to promote Guizhou's practice. Fourthly, a total of 170 million yuan of provincial forest reform funds have been invested, and about 16 billion yuan of social capital has been leveraged to build forest health industry clusters and 320 natural education bases. Fifthly, the province has planted 75900 hectares of flowers and seedlings, with annual sales of 1.622 billion yuan, 46 flower markets, 1072 flower enterprises, more than 2800 flower operators and nearly 60000 employees. Sixthly, we should cultivate 15 key national forestry leading enterprises, 266 provincial forestry leading enterprises, 2 national forestry industry demonstration parks and 30 national under-forest economic demonstration bases. Introduce "Guilin Loan" and "Industry Chain Finance" measures.

[Promoting Forestry by Science and Technology] Institute of Plateau Forestry, Chinese Academy of Forestry, Guizhou Province

Chen Xiaoming and Qiu Jiansheng of the Academy of Forestry Sciences won the second prize of Liangxi Forestry Science and Technology Award for Innovation and Application of Key Technologies for Efficient Cultivation and Industrialization of Galla Chinensis. Guizhou Normal University, Guizhou Baili Rhododendron Management Area Research Institute Li Chaochao and Wang Lingjun completed the Key Technologies for Cultivation and Renewal of Wild Rhododendron Forest in Guizhou, which won the third prize of Liangxi Forestry Science and Technology Award. Research on Background and Conservation Value of Biodiversity in 10 Nature Reserves in Guizhou, completed by Luo Yang and Deng Lunxiu of Guizhou Academy of Forestry Sciences, won the second prize of Guizhou Science and Technology Progress Award; Biological Control Technology of Batocera horsfieldi, Walnut Stem Borer in Guizhou, completed by Fu Fuyong and Situ Chunnan of Zunyi Forestry Research Institute, won the third prize of Guizhou Science and Technology Progress Award; Zhang Jiawei and Qiao Wenlang of Guizhou Geological Survey Institute and Fanjingshan World Natural Heritage Conservation Administration won the third prize of Guizhou Science and Technology Progress Award for their major innovation and application of Fanjingshan World Natural Heritage Geoscience. He won one second prize in the first National Forest and Grass Science Experiment Exhibition and one excellent prize in the first National Natural Education Creative Product Design Competition. One person was awarded the "National Advanced Worker in Forestry and Grass System" and the "Guizhou Youth Science and Technology Award". Aha Lake Wetland Park was awarded the first batch of popular science bases by the Ministry of Science and Technology and the State Forestry and Grassland Administration. Guizhou Chishui Bamboo Forest Ecosystem National Positioning Observation and Research Station has been approved to be newly built, the National Engineering Research Center for Processing and Comprehensive Utilization of Idesia polycarpa has passed the evaluation of the State Forestry and Grassland Administration, and the Guizhou Provincial People's Government has approved the establishment of Guizhou Eco-Energy Vocational College. New research platforms such as Fanjingshan Guizhou Golden Monkey Research Center, Rosa roxburghii Germplasm Resource Bank and Biodiversity Conservation Strategic Plant Resource Bank have been built. Twenty-four new forestry scientific research projects have been established, and 42 scientific and technological achievements of forestry and grass have been included in the scientific and technological extension achievement database of the State Forestry and Grassland Administration. It has been granted 24 national invention patents and 27 utility model patents, and has completed the acceptance of 43 projects. Ten provincial-level local standards for forestry in Guizhou Province, such as Forest Carbon Sequestration Monitoring and Measurement Method, have been approved, and eight provincial-level local standards in Guizhou Province, such as Technical Regulations for Sowing and Raising Seedlings of Idesia polycarpa, have been approved and issued. 2230 batches of quality and safety testing of edible forest products have been completed. For the first time, Rosa roxburghii seeds were carried out in space. Thirty-four demonstration projects have been implemented, 11 fine varieties, 48 new technologies and 34 new demonstration bases have been built.

[Forest and Grass Seedlings] Guizhou Forestry Bureau and Guizhou Science and Technology Department jointly formulated and promulgated the Guizhou Forest and Grass Seed Industry Development Plan (2022-2035). The Provincial Forestry Bureau has formulated and promulgated the Measures for the Management of Provincial Forestry Guaranteed Nurseries in Guizhou Province, identified 69 provincial forestry guaranteed nurseries, and explored the management mechanism of "provincial overall planning + municipal and state allocation + nursery implementation" for the production, management and free allocation of guaranteed seedlings. Innovative seedling allocation mechanism, the first in the country to implement the "national key forest seed base production of improved varieties of scions, storage of precious native tree seeds, protective seedlings" three free allocation, by the provincial and municipal forestry authorities free allocation of improved varieties and precious native tree seeds 13173 0.3 kg, allocation of 26.75 million protective seedlings. The large data platform of "Guizhou Forest Seedling" has been popularized and applied in an all-round way, and 1.087 billion seedlings of economic forests, ecological timber forests and landscape greening have been cultivated throughout the year. To improve the technical system of rapid identification of economic forest varieties at seedling stage, one set of technical system of identification of Zanthoxylum bungeanum and walnut varieties was established based on DNA barcoding, and two draft standards of "Technical Regulations for Identification of Walnut Varieties by SSR Molecular Markers" and "Technical Regulations for Identification of Zanthoxylum bungeanum Varieties by DNA Barcoding" were drafted. Twenty-five high-yield and high-quality forest and grass varieties, such as Zanthoxylum bungeanum, Camellia oleifera, Macadamia nut and Lotus corniculatus, were examined (identified), accounting for 86% of the total number examined (identified) during the 13th Five-Year Plan period.

[Forestry Reform] The first is to promote the reform of the management system of Maolan Nature Reserve. On June 4, 2023, the General Office of the Guizhou Provincial Committee and the General Office of the Provincial People's Government issued the Reform Plan for the Management System of Maolan National Nature Reserve. Second, deepen the reform of state-owned forest farms. On December 29, the provincial government office issued the "Pilot Implementation Plan for Exploring the Incentive Mechanism of Operating Income Distribution in State-owned Forest Farms in Guizhou Province", and carried out the pilot incentive mechanism of operating income distribution in four state-owned forest farms, such as Longli Forest Farm. We will implement the Plan for Deepening the Reform of Collective Forest Tenure System issued by the General Office of the Central Committee of the Communist Party of China and the General Office of the State Council, and study and draft the implementation plan for the reform in Guizhou Province. "Guizhou Province innovates six mechanisms to deepen collective forest reform" has been promoted by the State Forestry and Grassland Administration. Third, we will continue to promote the pilot project of forestry carbon sequestration. "From selling timber to selling air, opening up a new world of forestry carbon sequestration in Guizhou" won the first prize of the provincial organ reform and innovation project in 2023. Explore the working mechanism of linking forestry carbon sequestration with administrative law enforcement and criminal justice. On December 17, the Guizhou Provincial Higher People's Court, the Provincial Forestry Bureau and other six departments jointly issued the Opinions on the Subscription of Forestry Carbon Sequestration for Compensation for Eco-environmental Damage in the Case of Destruction of Forest Resources. The pilot project of forest carbon sink index insurance was evaluated as an excellent reform pilot project in Guizhou Province in 2023. The declared "Research on the Realization Path of Rights and Interests Trading of Forest Coverage Indicators in Guizhou Province" was selected as a major research topic of the CPC Guizhou Provincial Committee in 2023 to comprehensively deepen the reform and won the Excellent Reform Topic Award, which guided the Guiyang Municipal Government Office to formulate and issue the "Implementation of Horizontal Ecological Compensation Mechanism in Guiyang City to Promote the Stability of Forest Coverage". Guiyang Guanshan Lake District and Kaiyang County, Yunyan District, Nanming District and Xifeng County signed purchase agreements respectively, with a total area of 83.25 hectares and an amount of 1.9329 million yuan. Fourthly, we will continue to promote the pilot project of forest health base construction, and "Research on the Path of High-quality Development of Forest Health Industry in Guizhou Province" was awarded the third prize for outstanding achievements in the investigation and research of major issues of the Provincial Party Committee in 2023.

[Fund and Project Management] First, for the first time, Guizhou Province received a national subsidy of 6.02 million yuan for comprehensive monitoring of forests, grasslands, wetlands and deserts, and a subsidy of 0.1 billion yuan for reward expenditure for supervision and assessment of forest chiefs. Complete the declaration, provincial competitive evaluation and submission of the pilot demonstration project of land greening financed by the central government in 2023. To carry out the declaration, provincial competitive evaluation and submission of the Central Finance Camellia oleifera Industry Development Demonstration Award and Subsidy Project in 2023, and recommend the "Tongren Central Finance Camellia oleifera Industry Development Demonstration Award and Subsidy Project" to participate in the national competitive evaluation. The second is to take the lead in compiling and promulgating the Guidelines for the Implementation and Management of Investment Projects within the Central Budget for Ecological Protection and Restoration in Key Areas of Guizhou Province (Trial Implementation), so as to promote the standardized management and implementation of ecological protection and restoration projects in key areas of Guizhou Province. To compile and issue the Management Measures for the Completion and Acceptance of Forestry Capital Construction Projects in Guizhou Province (Interim) to solve the problems of insufficient basis and lack of standardized process guidance for forestry departments at all levels in the province when applying for the completion and acceptance of forestry capital construction projects. Third, we should innovate the work management mode and establish an information platform. Establish the department's internal budget performance management project database platform, based on the project database, run through the budget declaration, approval, implementation, performance declaration, monitoring and self-evaluation, standardize the project database and budget performance management by means of information technology, and provide basic technical support for the "531" project database. Fourth, accelerate the construction of budget expenditure standard system. Guizhou Forestry Bureau, as the only pilot department in the province arranged by the Provincial Department of Finance, has established a set of "Guizhou Forestry Bureau Department Overall Project Expenditure Budget Standard System", including 69 sets of system tables and basic element tables, formulated more than 1,500 element control (cost element) standards, and matched more than 2,000 core performance indicators.

[Voluntary tree planting] We continued to promote voluntary tree planting by five-level cadres. For nine consecutive years, on the first day of work after the Spring Festival, the main leaders of Guizhou Province participated in the voluntary tree planting activities by five-level cadres in provinces, cities, counties and villages. A total of 1469 voluntary tree planting sites were set up in the province, with 138,000 participants and 1.18 million trees planted, covering an area of 0.12 million hectares. A total of 54.41 million trees (including conversion) were planted voluntarily throughout the year, and 14.4 million people participated in voluntary tree planting.

The Standing Committee of the CPC Guizhou Provincial Committee and the Standing Committee of the Guizhou Provincial People's Government heard a special report on the work of the forest chief system, and the provincial ecological environment protection conference emphasized the deployment of the work of the forest chief system. Provincial-level chief foresters were requested to issue the Decree on Strengthening the Protection of Forest and Grassland Resources, and the list of provincial-level chief foresters was updated in a timely manner. A total of 46913 chief foresters were set up in provinces, cities, counties and villages, and 97 chief foresters at all levels were issued. Provincial forest chiefs took the lead in carrying out forest patrol activities, with a total of 594000 forest patrols in the province, coordinating and solving 2625 key and difficult problems. On June 18, Xu Lin, Secretary of the CPC Guizhou Provincial Committee, Chairman of the Standing Committee of the Guizhou Provincial People's Congress and Chief Forest Officer of the province, went to Changpoling National Forest Park to carry out forest patrol activities. On June 19, Deputy Secretary of the CPC Guizhou Provincial Committee, Governor of the People's Government of Guizhou Province and Chief Forest Officer of Guizhou Province went to Ganxi Forest Farm in Guiding Li Bingjun to carry out forest patrol activities. Luo Qiang, Vice Governor of Guizhou Provincial People's Government, presided over the provincial meeting to complete the inspection of the national forest chief system in 2023. The provincial forest chief office carried out the 2022 annual assessment of the forest chief system in 9 cities (prefectures) and 88 counties (cities and districts) of the province. The results of the assessment were approved by the provincial chief forest chief and notified to the Party committees and governments of the cities (prefectures), counties (cities and districts). On May 4, Jiangkou County won the incentive of the State Council's forest chief system in 2022.

[Theme Forum of Guiyang International Forum on Ecological Civilization 2023 "Protecting the Beauty of Nature, Promoting Green Development-Promoting the Construction of Nature Reserve System with High Quality"] On July 8, the theme forum was held in Guiyang. Yan Zhen, deputy director of the State Forestry and Grassland Administration, Yin Weilun, academician of the Chinese Academy of Engineering, attended the forum, and Wang Zhong, deputy director of the Standing Committee of the Guizhou Provincial People's Congress, delivered a speech. A total of 252 guests were invited to the sub-forum, including representatives of the United Nations Development Programme (UNDP), the World Wide Fund for Nature (WWF), the World Conservation Union (IUCN) and other international organizations, as well as well-known experts and scholars from the Development Research Center of the State Council, the Chinese Academy of Sciences, Peking University and the Central Radio and Television Station. Leaders of forestry and grassland authorities in all provinces (autonomous regions and municipalities) and representatives of some national nature reserve management agencies have achieved full coverage in the five fields of government, industry, education, research and media, and promoted exchanges and cooperation in policy, system and technology in the field of nature reserves throughout the country. The sub-forum issued the Guiyang Declaration, which was the strongest voice for the construction of China's nature reserve system, and received multi-dimensional publicity and reports from the front page of People's Daily, Xinhua Daily Telegraph, CCTV Morning News, Focus Interview, National Geographic and other authoritative media.

[Forest Quality Improvement] Guizhou Forestry Bureau issued "Detailed Rules for the Implementation of Forest Tending in Guizhou Province", "Technical Guidelines for the Construction of National Reserve Forests in Guizhou Province (Trial Implementation)", "Measures for the Construction and Management of National Reserve Forests in Guizhou Province (Trial Implementation)", "Implementation Plan for Promoting the Construction of National Reserve Forests with High Quality in Guizhou Province", and compiled "Guizhou Province". Promote the construction of forest quality improvement projects such as national reserve forests with high quality. The National Pilot Work Program for Sustainable Forest Management in Guizhou Province in 2023 was issued to continuously promote the pilot work of sustainable forest management and complete the pilot construction of 0.1 million hectares. To carry out pilot demonstration of tree species structure adjustment, nine pilot units, such as Qingzhen, Chishui, Longli and Yinjiang, were identified, and 26700 hectares of pilot construction of tree species structure adjustment were completed. Jiangkou County, Guizhou Province, approved the "Whole County Promotion Plan for Adjusting Tree Species Structure and Improving Forest Quality", and supported Jiangkou County to become the first county in the province to promote the adjustment of tree species structure and improve forest quality. We will support the construction of the project to precisely improve the forest quality of the forest belt around the city of Guiyang, and help the strategic development of the "strong provincial capital". In 2023, the province implemented a precise improvement of 315,200 hectares of forest quality. Among them, there are 47,300 hectares of forest tending, 134,300 hectares of low-yield and low-efficiency forest transformation, and 133,600 hectares of degraded forest restoration.

[Audit work] The first is to complete 17 internal audit projects, including the 2022 budget execution and financial revenue and expenditure audit of 11 units directly under Guizhou Forestry Bureau, the economic responsibility audit of the principal responsible persons of 2 units directly under Guizhou Forestry Bureau, and 4 special audits. In 2022, the budget execution and financial revenue and expenditure audit of five directly affiliated units and the economic responsibility audit of the principal responsible persons of five directly affiliated units found problems and rectified them, thus achieving full coverage of the audit of the directly affiliated units of the Bureau. The second is to combine internal audit with internal inspection and discipline inspection, to combine internal audit with discipline inspection and inspection, to form a joint force, to cooperate with the completion of the internal inspection work of three units and the review of economic issues of relevant personnel, and to promote the coordination of various supervision. Thirdly, we will continue to take the lead in carrying out the rectification and implementation of the problems found in the relevant national and provincial audits, inspections, inspections and inspections. Fourth, cooperate with the Kunming Special Office of the Audit Office to carry out the audit of forest and grass funds in Guizhou Province from 2021 to 2023.

[Establishment of National Park] Guizhou Forestry Bureau carried out legislative research on Fanjingshan National Park Regulations, relying on Wuling Landscape Restoration Project in Tongren City, and started the construction of ecological corridors. Complete the monitoring and habitat investigation of Guizhou golden monkey and the investigation of the current situation of Fir resources in Fanjingshan. Do a good job in publicity and promotion of Fanjingshan National Park, shoot promotional films and feature films of Fanjingshan National Park, and produce promotional brochures. Establish Fanjingshan Regional Party Building Alliance, carry out the redemption of artificial commercial forests, and promote community co-management and co-construction. On July 25, the State Forestry and Grassland Administration gave feedback on the preliminary examination of the materials for the establishment of Fanjingshan National Park. On September 1, according to the feedback from the State Forestry and Grassland Administration, the materials for the establishment of Fanjingshan National Park were revised and improved, and the Request for Assessment of the Establishment of Fanjingshan National Park in Guizhou Province was submitted to the State Forestry and Grassland Administration, and the application for review was submitted and the revised and improved materials were submitted. Fanjingshan National Park has now entered the stage of "effectiveness evaluation". On May 23, the Forestry Bureau of Guizhou Province and the Forestry Bureau of Guangxi Zhuang Autonomous Region jointly reported the Plan for the Establishment of Southwest Karst National Park. On October 23, the State Forestry and Grassland Administration gave feedback on the review of the Southwest Karst National Park Establishment Plan. On December 7, the people's governments of the two provinces (regions) jointly reported to the State Forestry and Grassland Administration the Plan for the Establishment of the Southwest Karst National Park and submitted an application for the establishment of the Southwest Karst National Park.

Forestry Yearbook of Guizhou Province in 2022

[Overview] In 2022, Guizhou Forestry Bureau comprehensively studied and implemented the spirit of the 20th National Congress of the Communist Party of China, and thoroughly implemented the spirit of General Secretary 's important speech on inspecting Guizhou. Grasp the opportunity of "Opinions of the State Council on Supporting Guizhou to Break a New Path in the Development of the Western Region in the New Era" (Guofa [2022] No.2) and "Several Measures to Support the High-quality Development of Forestry and Grassland in Guizhou" issued by the State Forestry and Grassland Administration, and adhere to the modernization requirements of harmonious coexistence between man and nature. The province has completed 183,300 hectares of afforestation, 23,000 hectares of grassland ecological restoration, 725 square kilometers of comprehensive control of rocky desertification, 11.1111 million hectares of forest area, 62.81% of forest coverage, 88.9% of grassland comprehensive vegetation coverage, and 67% of grass and livestock balance. It has completed the construction of 194700 hectares of characteristic forestry industrial base, the area of under-forest economic management and utilization of forest land has reached 1973300 hectares, the construction of 123000 hectares of national reserve forest, and the financing loan of 16.2 billion yuan for the national reserve forest project. The total output value of forestry industry in the province has exceeded 400 billion yuan. The national and provincial forest chief system has been inspected and assessed, and the forest chief system has been upgraded to the first-level index of performance evaluation and assessment for promoting high-quality development in cities and counties.

[Land Greening] Carry out ecological civilization education and voluntary tree planting activities, and continue to carry out voluntary tree planting activities for five-level cadres. Prepare for the assessment of the suitability of afforestation and greening space, and realize the accurate mapping of afforestation tasks. Three national forest cities, one forest city, 52 forest townships, 181 forest villages, 904 forest households and 30 forest villages with beautiful landscape have been built in Guizhou Province. Rural greening and beautification were carried out, and the coverage rate of village greening in the whole province reached 46.24%. Adhering to the principle of "forest is suitable for forest and grass is suitable for grass", 183,300 hectares of afforestation and 23,000 hectares of grassland ecological restoration were completed throughout the year.

[Forestry Resources Protection] The national and provincial forest chief system supervision and assessment has been completed, and the forest chief system has been upgraded to the first-level index of performance evaluation and assessment for promoting high-quality development in cities and counties, and five forest chief system theme parks have been built. Fully implement the task of forest resources management and protection, continue to stop commercial logging of natural forests, and the rate of stopping logging and management of natural commercial forests reaches 100%. The social insurance subsidy policy for the Natural Forest Protection Project has been conscientiously implemented, and the participation rate of employees of forestry enterprises and institutions in forest areas has reached 100%. A total of 182,800 ecological forest rangers have been accurately recruited and re-employed, and the management and protection of forest and grass ecological resources have been gridded and fully covered. In 2021, the rectification rate of forest inspection cases reached 100% for the first time in the country. In 2022, 714 cases of wildlife were investigated and dealt with, and 43 cases of illegal use of forest land in three types of projects, including wind farms, photovoltaic power stations and non-coal mines, were investigated and dealt with. We will continue to carry out the second round of central ecological environment protection supervision, provincial ecological environment protection supervision and rectification of forest-related issues, and the "Green Shield" nature reserve supervision and inspection. In 2022, the illegal cases of forest land and trees in the whole province decreased by 34% compared with the same period last year. Forestry is included in the "one-window operation" according to the application items. Establish a reporting system for the advance use of forest land for key infrastructure projects at or above the provincial level, entrust the provincial examination and approval authority for the use of forest land for construction projects to municipalities and prefectures for implementation, strive for an additional 3186 hectares of forest land quota from the state, and examine and approve the use of 11000 hectares of forest land. The Interim Measures for the Connection between Forest and Grass Administrative Law Enforcement and Criminal Justice in Guizhou Province and the Measures for the Delimitation and Management of Local Public Welfare Forests in Guizhou Province were promulgated. Complete the transfer of forest vegetation restoration fees and grassland vegetation restoration fees to the tax authorities for collection. Complete the comprehensive monitoring of forest and grass moisture. Five national wetland parks have passed the acceptance test and completed the dynamic monitoring of 42 important wetlands at the provincial level. The first batch of 12 provincial-level terrestrial wildlife epidemic disease monitoring stations, including Guiyang and Huaxi in Guizhou, were announced. Start the construction of laboratory for preliminary inspection of epidemic foci and diseases of wild animals. Start the investigation of new national key protected wild plants. The first biodiversity survey of large plots of forest ecosystems was launched. The establishment of Fanjingshan National Park has been approved, the Southwest Karst National Park has been incorporated into the National Park Spatial Layout Plan, and the provincial nature reserves have been integrated and optimized into the achievements of "three districts and three lines". Huangguoshu Scenic Spot was selected into the Green List of the World Conservation Union.

[Forest pest control] Explore the "sky-air-ground" integrated monitoring system of pine wood nematode disease. For the first time, a general survey of alien invasive species of forest and grass was carried out. The Regulations on the Prevention and Control of Forestry Pests in Guizhou Province were formally implemented, and the Plan for Improving the Prevention and Control Ability of Forest and Grass Pests in Guizhou Province (2023-2025) was issued. 75 cases of forestry pests were investigated and dealt with throughout the year. Two pine wood nematode epidemic areas, Leishan and Libo, were successfully removed. The disaster rate of forest pests in the whole year decreased by 40.8% compared with the same period last year, far below the national control target.

[Forest Fire Prevention] For the first time, the provincial government issued the "Guizhou Province Forest and Grassland Fire Ban". The system of fire prevention interview, fire information and verification management was introduced, and 278 people were held accountable. Cooperate to complete the work of Heilongjiang Forest Fire Brigade stationed in Guizhou Province. We will further promote actions such as special rectification of forest fire prevention and control and "cracking down on non-violations" in the forestry field. There were 9 forest fires in the whole year, the damaged forest area was 63.17 hectares, the forest fire damage rate was 0.0057 ‰, and the forest fire damage rate was far below the national control index.

[Forestry industry] The total output value of forestry industry in the province has exceeded 400 billion yuan, with 254 provincial leading forestry enterprises and 15 national key leading forestry enterprises. The area of under-forest economic management and utilization of forest land in the whole province is 19733000 hectares. There are 337,300 hectares of bamboo, 246,000 hectares of Camellia oleifera, 116,700 hectares of pepper, 68,000 hectares of honey locust, 140,000 hectares of Rosa roxburghii and 252,700 hectares of walnut. The development scale of the three industries of Gleditsia sinensis, Rosa roxburghii and Chimonobambusa quadrangularis ranks first in the country. 46700 hectares of Camellia oleifera base have been completed. Completed the construction of 94,700 hectares of characteristic forestry industrial base, the transformation of 8,000 hectares of Rosa roxburghii inefficient forest, and the transformation of 23,300 hectares of fungus timber forest. Ten people's livelihood facts of the province have been completed (the task of upgrading the forest health trail by 100 kilometers), and the total area of the forest health base in the province has reached 86,000 hectares. There are 20 provincial-level natural education bases. The capital in place for investment attraction is 9.1 billion yuan. To compile the Evaluation Index System of Modern Forestry Industry Demonstration Zone in Guizhou Province and the 14th Five-Year Plan of Modern Forestry Industry Demonstration Zone in Guizhou Province, and start the construction of modern forestry industry demonstration zone. The Action Plan for the Development of Idesia polycarpa Industry in Guizhou Province has become the first province in China to promote the development of Idesia polycarpa industry from the provincial level, and has successfully registered the characteristic trademark of "Guixiansen" Idesia polycarpa oil.

The first Key Laboratory of Biodiversity Conservation in Southwest Karst Mountains of the State Forestry and Grassland Administration was listed, and the provincial forestry system won one second prize and three third prizes of the Provincial Government Science and Technology Progress Award. The state-owned Longli Forest Farm in Guizhou Province and Changpoling Forest Farm in Guiyang City are listed as the third batch of national forest germplasm resource banks. Complete "Technical Regulations for Seedling Raising of Idesia polycarpa" and "Technical Guidelines for Cultivation and Management of Idesia polycarpa (Trial)". The Measures for the Management of Provincial Forestry Guaranteed Nurseries were issued, 24 guaranteed nurseries were built, 25 improved varieties of forest and grass such as Camellia oleifera were examined (recognized), and 4 new germplasm resource banks at or above the provincial level were added. The DNA molecular identification technology system of walnut and Zanthoxylum bungeanum seedlings in Guizhou Province was established. Nine local standards, such as the Standards for the Construction of Biological Fire Prevention Isolation Zones in Guizhou Province and the Standards for the Construction of Forest Health Walk, have been set up. Three local standards, including Classification and Code of Vascular Plants in Guizhou and Technical Regulations for Cultivation of Red Camellia in Guizhou, have been approved and issued. Promote 40 advanced and practical forestry scientific and technological achievements and standards, and build 38 demonstration bases for forestry science and technology extension. There are 89 scientific research projects. Guizhou Academy of Forestry Sciences was selected as the first batch of national popular science education bases, and Chishui Alsophila National Nature Reserve and Fanjingshan World Natural Heritage Site were selected as the first batch of national popular science bases for natural resources. The state-owned Longli Forest Farm in Guizhou Province and Changpoling Forest Farm in Guiyang City are listed as the third batch of national forest germplasm resource banks.

[Forestry Reform] promulgated the Rules for the Application of "No Punishment for the First Violation" and Inclusive Exemption for Minor Illegal Acts in Forestry of Guizhou Province (Trial Implementation), and the List of "No Punishment for the First Violation" and Inclusive Exemption for Minor Illegal Acts in Forestry of Guizhou Province (Trial Implementation). Establish the reform system of "one law, one case and one list". To formulate the Format Documents, Filling Instructions and Model Documents of Administrative Penalties for Forestry System in Guizhou Province, and standardize 35 kinds of administrative penalty documents for forestry. Sixteen county-level forestry administrative authorities were granted to economically developed towns, and 13 county-level forestry administrative authorities were granted to towns and streets. The Guiding Opinions on the Pilot Project of Exploring the Incentive Mechanism for Operating Income Distribution of State-owned Forest Farms in Guizhou Province were issued. "Exploration of the Path of Modernization, Transformation and Development of State-owned Forest Farms in Guizhou" won the third prize for outstanding achievements in the investigation and research of major issues of the Guizhou Provincial Committee. It is the first province in China to incorporate forest management units that independently compile forest management plans into the pilot reform of cutting quota management by issuing the Work Program for the Five-year General Control Reform of the Main Cutting Quota of Artificial Commercial Forests in Guizhou Province. The Notice of the Provincial Forestry Bureau on Implementing the Reporting System for the Advance Use of Forest Land in Key Infrastructure Projects at or above the Provincial Level was issued. We will continue to promote the pilot projects of forest management, pledge loans for compensation income rights of public welfare forests, redemption reform of commercial forests in key ecological areas, and redemption reform of commercial forests in key ecological areas, which have been approved by the main leaders of the State Forestry and Grassland Administration. The Action Plan for High-quality Development of Forestry Carbon Sequestration in Guizhou Province and the Technical Guidelines for Measurement and Monitoring of Forest Carbon Sequestration in Guizhou Province (Trial Implementation) were issued. The contracted area of forestry carbon sequestration (carbon ticket) projects in the province is 614700 hectares, the development area is 36000 hectares, 8 forestry carbon tickets are developed, 508 million yuan of forestry carbon sequestration (carbon ticket) credit, 173 million yuan of loan and 4.98 million yuan of transaction amount are completed.

[Strong basic guarantee] 24.6 billion yuan of forestry investment was completed in the whole year, and a project database platform for budget performance management of forestry system in Guizhou Province was established. Actively promote the construction of provincial ecological and energy vocational colleges. Revise the Conditions for the Evaluation of Forestry Professional Titles in Guizhou Engineering Series and the Measures for the Management of the Evaluation of Forestry Professional Titles in Guizhou Forestry Engineering Series, and promulgate the Key Points for the Allocation of Section-level Cadres in Guizhou Forestry Bureau, the Measures for the Management of News and Publicity in Guizhou Forestry Bureau, the Measures for the Management of Information Construction in Guizhou Forestry Bureau (Trial Implementation), and the Measures for the Assessment of Forestry Party and Government Information. It compiled 12 issues of Guizhou Forestry Publicity and Reporting Collection, held the first Plenary meeting of the Organizing Committee of Guizhou Forest Concern Activities, and established two green camps for national youth natural education. Successfully held the "Thanksgiving Endeavor New Journey Green Water and Green Mountains to See Guizhou" Poetry Competition, with more than 6000 entries. More than 2300 pieces of forestry information were publicized and reported in provincial media throughout the year.

[The voluntary tree planting activity of the whole province will be held in 2022] On February 7, 2022, the voluntary tree planting activity of the whole province will be held simultaneously at five levels of provinces, cities, counties and villages. Chen Yiqin, secretary of the CPC Guizhou Provincial Committee and director of the Standing Committee of the Provincial People's Congress, Li Bingjun, deputy secretary of the CPC Guizhou Provincial Committee and governor of the province, Liu Xiaokai, chairman of the CPPCC Provincial Committee, Lan Shaomin, deputy secretary of the CPC Guizhou Provincial Committee and deputy director of the Standing Committee of the Provincial People's Congress, came to Xianglu Mountain, Gaoxue Village, Dongfeng Town, Wudang District, Guiyang City, to participate in voluntary tree planting activities with cadres and ma sses, and to work together to make Guizhou green and to build ecological civilization. Members of the Standing Committee of the Guizhou Provincial Committee, the Standing Committee of the Provincial People's Congress, the Provincial Government, the Leading Group of the Provincial Political Consultative Conference and the Party Group, the Guizhou Military Region, the Provincial Court, the Provincial Procuratorate and the Provincial Armed Police Corps are mainly responsible for comrades participating in tree planting activities at provincial voluntary tree planting sites.

[The State Forestry and Grassland Administration issued "Several Measures to Support the High-quality Development of Forestry and Grass in Guizhou"] On April 12, 2022, the State Forestry and Grassland Administration issued "Several Measures to Support the High-quality Development of Forestry and Grass in Guizhou", which clearly supported the high-quality development of forestry and grass in Guizhou from seven aspects. First, we should scientifically promote land greening. The second is to strengthen the protection and management of forest and grass resources. The three is to promote the construction of a nature reserve system with national parks as the main body. Fourth, enhance the ability of disaster prevention and mitigation. Fifth, explore and carry out pilot forest and grass reform. Sixth, to promote the high-quality development of forest and grass industry with distinctive advantages. Seventh, we should strengthen scientific and technological research and personnel training.

[The establishment of Fanjingshan National Park was approved] On April 25, 2022, the National Park Administration sent a letter to the People's Government of Guizhou Province, "Letter on Approving the Establishment of Fanjingshan National Park" (Park Letter [2022] No.3), approving the establishment of Fanjingshan National Park in Guizhou Province. On October 31, the Forestry Bureau of Guizhou Province submitted to the National Park Administration the "Request for Assessment of the Establishment of Fanjingshan National Park in Guizhou Province", "Comprehensive Scientific Investigation Report of the Assessment Area of Fanjingshan National Park", "Conformity Confirmation Report of Fanjingshan National Park", "Social Impact Assessment Report of the Establishment of Fanjingshan National Park". The work of "carrying out creation" has been basically completed, and the results are obvious.

[The implementation of the forest chief system has achieved remarkable results] Timely update the list of provincial forest chiefs. Supporting systems such as "Key Points for the Work of Forest Chief System in Guizhou Province in 2022", "Guizhou Ecological Day" Forest Chief Patrol Activity Program in 2022 "," Implementation Plan for the Assessment and Monitoring of Forest Chief System in the Evaluation of High-quality Development Performance of Cities and Counties "were issued. Forest chiefs at all levels issued 96 forest chief orders, carried out 718000 forest patrols, and provincial, municipal and county-level forest chiefs helped solve 364 key and difficult problems. Five forest chief system theme parks have been built. The forest chief system has been incorporated into the high-quality performance evaluation index of cities and counties, and the forest chief system has entered the training classroom of the Party School of the Provincial Party Committee. Jiangkou County was awarded the State Council's forest chief system supervision and encouragement.

[Fully promote the development of under-forest economy] Work programs such as "Key Points for High-quality Development of Under-forest Economy in the Province in 2022" and "Work Program for Improving Quality and Efficiency of Under-forest Economic Development in Guizhou Province" were issued, and the "Special Statistical Investigation System for Under-forest Economy in Guizhou Province (Trial Implementation)" was promulgated. The area of under-forest economic management and utilization of forest land in the province reached 1.9733 million hectares, and the output value of the whole industrial chain was 60.5 billion yuan. The development of under-forest economy was included in the performance evaluation of cities and counties promoting high-quality development in 2022. Guizhou Forestry Bureau, Guizhou Investment Promotion Bureau and Guangdong Forestry Bureau co-sponsored the Shenzhen-Guizhou Forestry Industry Investment Invitation Activity in Shenzhen in 2022. "Research on Statistical Monitoring of Undergrowth Economic Development in Guizhou Province" won the Excellent Award for Major Issues Investigation and Research of Guizhou Provincial Committee.

[In-depth implementation of forest quality improvement] "Three-year Action Plan for Accurate Improvement of Forest Quality in Guizhou Province (2023-2025)", "General Plan for Demonstration Forest Construction of Pilot Demonstration Project for Adjusting Tree Species Structure and Improving Forest Quality in Guizhou Province", "Monitoring Plan for Pilot Demonstration Project for Adjusting Tree Species Structure and Improving Forest Quality in Guizhou Province", "Provincial Forestry Bureau explores the pilot work of optimizing and adjusting tree species structure of artificial commercial pure forest" Enter the Plan. The pilot demonstration of tree species structure adjustment was carried out, and six pilot counties, Xishui, Zhijin, Jiangkou, Yuping, Congjiang and Huishui, were identified. Twenty thousand hectares of tree species structure adjustment have been completed, and Jiangkou County has explored the model of "national reserve forest + tree species structure adjustment", which has set an example for Guizhou Province. In 2022, Guizhou Province implemented the precise improvement of forest quality of 274,500 hectares, including 40,800 hectares of forest tending, 144,500 hectares of low-yield and low-efficiency forest transformation, 89,100 hectares of degraded forest restoration, and 4,600 hectares of timber strategic reserve base construction.

China Forestry Yearbook Guizhou Province 2021 Materials

[Overview] In 2021, Guizhou Forestry Bureau thoroughly implemented 's socialist ideology with Chinese characteristics in the new era and the spirit of General Secretary 's important speech on inspecting Guizhou. In accordance with the requirements of systematic management of mountains, rivers, forests, fields, lakes and grasses, it adhered to the overall situation of high-quality development and accomplished various objectives and tasks. The province has completed 240700 hectares of afforestation, 20.46 million hectares of grassland ecological restoration, 640 square kilometers of comprehensive control of rocky desertification, 62.12% of forest coverage, 88.5% of grassland comprehensive vegetation coverage, and 878.3 billion yuan of forest ecological service value per year. The construction of characteristic forestry industry base has reached 194700 hectares, the area of forest land for economic utilization under forest has reached 1866700 hectares, the construction of national reserve forest has reached 143300 hectares, and the total output value of forestry industry in the province has reached 371.9 billion yuan. The five-level forest chief system has been constructed in an all-round way.

Land greening carried out a series of activities for the 40th anniversary of national voluntary tree planting, and continued to carry out voluntary tree planting activities for five-level cadres. One provincial forest city, 85 forest townships, 223 forest villages and 1345 forest households have been built. Rural greening and beautification were carried out, and the coverage rate of village greening in the whole province reached 44.22%. Adhering to the principle of "forest is suitable for forest and grass is suitable for grass", the province has completed afforestation of 240700 hectares and grassland ecological restoration of 20460 hectares.

The total output value of forestry industry in the province reached 371.9 billion yuan, and the number of leading forestry enterprises at the provincial level reached 254. The area of under-forest economic development in the province reached 1.8667 million hectares, and the processing conversion rate reached 55.3%. Bamboo, Camellia oleifera, Zanthoxylum bungeanum and Gleditsia sinensis have been newly built and cultivated for 194700 hectares, walnut for 17300 hectares and fungus forest for 23300 hectares. Carry out a general survey of the current situation and development potential of forest resources. Build a characteristic forestry industry experience center and information release platform.

A total of 1628 administrative licenses for the use of forest land for construction projects have been handled in the province, with an area of 8757.8238 hectares approved for use and a forest vegetation restoration fee of 1.3 billion yuan levied. Continuing to carry out forest supervision and "six strict prohibitions" actions, the Guiyang Commissioner's Office of the United Nations National Forestry and Grassland Administration jointly listed and supervised four forest-related illegal cases in the province, which had a significant impact and lagged behind the progress of investigation and rectification. The transfer rate of forest-related criminal cases in previous years was 100%, and the execution rate of forest-related administrative cases in previous years was 96.18%. A joint operation code-named "Qingfeng Action" was launched to crack down on illegal wildlife trade, 103 wildlife cases were investigated and 115 criminals were dealt with. The provincial forest supervision database and archives have successfully passed the national acceptance. Start the fourth rocky desertification survey in the whole province. The Measures for Compensation for Personal and Property Damage Caused by Terrestrial Wildlife in Guizhou Province were promulgated. The overall planning of four provincial scenic spots, Guanling Huajiang Grand Canyon, Panzhou Dadong Bamboo Sea, Qinglong 24 Road Guai and Zunyi Loushan, has been approved by the Guizhou Provincial People's Government.

Guizhou Provincial People's Government organized a teleconference on the prevention and control of pine wood nematode disease in Guizhou Province. The Regulations on the Prevention and Control of Forestry Pests in Guizhou Province were deliberated, adopted and promulgated at the 28th meeting of the Standing Committee of the 13th People's Congress of Guizhou Province. The General Plan for the Five-year Action for the Prevention and Control of Pine Wood Nematode Disease in Guizhou Province (2021-2025) has been compiled, and the area of infected trees of 0.13 million hectares has been completed throughout the year. Libo County and Leishan County passed the examination of the State Forestry and Grass Administration for the removal of epidemic areas. Guizhou Forestry Bureau, together with Tongren Municipal People's Government, carried out a special survey of pine wood nematode disease in the surrounding areas of Fanjing Mountain. The disaster rate of forest pests in the whole year was 0.191 ‰, which was lower than the national control index of 3 ‰.

A total of 9 forest fires occurred in the province, with a total area of 129.96 hectares and a damaged forest area of 48.94 hectares. The damage rate of forest fires was 0.0045 ‰, which was far below the national control index of 1 ‰ and the provincial control index of 0.8 ‰. No major forest and grassland fires, casualties and safety accidents occurred.

Forestry reform and the establishment of the five-level forest chief system in the whole province have been completed. Measures for Supporting the Transfer of Collective Forest Land Management Rights by Social Capital such as Industrial and Commercial Enterprises and Guiding Opinions on Accelerating the Pledge Loan of Compensation Income Rights for Public Welfare Forests in Guizhou Province were issued, and the Work Program for the Pilot Reform of Redemption of Artificial Commercial Forests in Key Ecological Areas of Guizhou Province (2021-2025) was issued jointly with the Guizhou Provincial Finance Department. Complete the pilot work of optimizing and adjusting local public welfare forests in Sandu County and Jinping County. The processing time limit for forestry examination and approval items has been reduced from 20 working days to 5 working days, and the processing time limit has been reduced by more than 70%. Forestry examination and approval items in the whole province can be handled through online application and mailing, so as to achieve zero running of applicants. All 98 items of administrative power applied for by the forestry system are included in the list of "province-wide and one-time" items, and the compilation of "province-wide and one-time" guidelines, key points of review and format texts are organized and entered into the province-wide system platform.

The "National Long-term Scientific Research Base of Pinus massoniana in Plateau Mountains" has been approved by the State Forestry and Grassland Administration. The Key Technology of Ecological Cultivation of Dendrobium candidum with Wild Trees of Guizhou Academy of Forestry Science was awarded the third prize of Guizhou Technological Invention; the Guiyang Forestry Informatization Project (Phase II) of Guiyang Forest Resources Management Station was awarded the third prize of Guizhou Science and Technology Progress. Five local standards, such as "Technical Regulations for Potted Flower Cultivation of Paphiopedilum" and "Technical Regulations for Seedling Cultivation of Loropetalum chinense", have been approved and issued, and the forestry industry standard of "Technical Regulations for Palm Cultivation" has been approved and issued. The state-owned Longli Forest Farm and Libo Huangjiang River National Wetland Park in Guizhou Province were named the fifth batch of "National Forest and Grass Science Popularization Base". Chishui Alsophila Spinulosa National Reserve Administration was awarded "Top Ten Popular Science Education Bases in Guizhou Province". Twenty-six training courses on forestry technology were held to explore a new mode of "online and offline" science popularization, with 126,000 people trained online.

The State Forestry and Grassland Administration and the People's Government of Guizhou Province have formed the Memorandum of Visit to Guizhou Province. We will carry out the adjustment of internal institutions, add the Development Planning Research Office and the Forest Chief System Office, abolish the Wetland Office and the Disaster Prevention Office, and adjust the establishment of the Finance Office. The Party Group of Guizhou Forestry Bureau of the Communist Party of China issued the Implementation Measures for Promoting Leading Cadres to Be Able to Go Up and Down (Trial Implementation) and the Implementation Opinions of the Party Group of Guizhou Forestry Bureau of the Communist Party of China on Further Encouraging Cadres to Take Responsibility and Correcting Errors (Trial Implementation). A total of 541 pieces of forestry information were reported in the national media and more than 1800 pieces in the provincial media. The press release room of Guizhou Forestry Bureau was set up, five press conferences were held, and one online interview was recorded by Guizhou Provincial People's Government Network. Publishing forestry books such as Forest Nature Education Guide, Guizhou State-owned Forest Farm and Guizhou Forest Park. Guizhou forestry network monitoring system was established, and 12 public opinion monitoring and analysis reports were formed.

[2021 provincial voluntary tree planting activities held] On February 18, 2021, the provincial voluntary tree planting activities were held simultaneously in provinces, cities, counties and villages. Chen Yiqin, Secretary of the Guizhou Provincial Party Committee and director of the Standing Committee of the Provincial People's Congress, Li Bingjun, Deputy Secretary of the Provincial Party Committee, Liu Xiaokai, chairman of the Provincial Political Consultative Conference, and Lan Shaomin, Deputy Secretary of the Provincial Party Committee, came to Xiaobi Village, Xiaobi Township, Shuanglong Airport Economic Zone, Guiyang City, to work directly with the cadres and masses to add green to the land of Guizhou and promote the construction of ecological civilization in the whole province. Members of the Standing Committee of the Guizhou Provincial Committee, the Standing Committee of the Provincial People's Congress, the Provincial Government, the Leading Group of the Provincial Political Consultative Conference and the Party Group, the Provincial Military Region, the Provincial Court, the Provincial Procuratorate and the Guizhou Provincial Armed Police Corps are mainly responsible for comrades participating in tree planting activities at the provincial voluntary tree planting sites.

On July 11, 2021, the theme forum of the 2021 Guiyang International Forum on Ecological Civilization "The Way of Forest Health and China" was held. The forum is co-sponsored by the State Forestry and Grassland Administration and the People's Government of Guizhou Province, and is hosted by the Forestry Reform Department of the State Forestry and Grassland Administration, Guizhou Forestry Bureau, Guizhou Forestry Industry Federation, Guizhou Brand Building Promotion Association, Guiyang Forestry Bureau and Beijing Zhongyi Dingshi Culture and Technology Co., Ltd. At the forum, Wu Shenghua, Vice Governor of Guizhou Province, and Liu Dongsheng, Deputy Director of the State Forestry and Grassland Administration, delivered speeches. Yang Chao, Chief Economist and Director of Forestry Reform Department of the State Forestry and Grassland Administration, delivered a keynote speech on the topic of "Developing Forest Health and Serving Public Health". Liu Average, Consultant Chairman of the Brand Evaluation Technical Committee of the National Standardization Organization, Chairman of the China Brand Construction Promotion Association and former owner of the National Standards Committee, and Will McGoldrick, Managing Director of the Nature Conservation Association (TNC) in the Asia-Pacific region, through video, Professor An Lizhe, President of Beijing Forestry University, Chen Houqi, Dean of Shanghai College of Precision Functional Medicine and Vice President of American Academy of Natural Medicine, made keynote speeches. The forum made five achievements: the first is to release the "Value of Forest Ecosystem Services in Guizhou Province in 2020", and the second is to release the "Group Standards of China Forestry Industry Federation-Standards for the Construction of Forest Health Towns and Standards for the Construction of Forest Health Homes". The third is to establish the Forest Health Research Institute of Guizhou Province, the Forest Health Medical Engineering Research Center of Guizhou Province, and the Forest Health Innovation Alliance of the Forestry Industry Federation of Guizhou Province, and issue the license; the fourth is to launch the "Construction Bank Shanrong Business Platform Guizhou Forest Health Zone"; the fifth is to read out the "Forest Health Guiyang Memorandum" by Hu Hongcheng, the Party Secretary and Director of the Forestry Bureau of Guizhou Province.

[Full implementation of the forest chief system] The Secretary of the Guizhou Provincial Party Committee and the governor of Guizhou Province serve as the "double general forest chief", 20 provincial leaders serve as the provincial forest chief, 18 provincial departments serve as the member units of the joint meeting, 50170 forest chiefs at the five levels of provinces, cities, counties and villages, and the five-level forest chief system is fully established. The implementation plan for the comprehensive implementation of the forest chief system in Guizhou Province and related supporting systems were issued, and cooperative mechanisms such as "forest chief + chief procurator" and "forest chief + small forest chief" were established, so as to basically establish a forest chief management system with cooperation between upper and lower levels, clear powers and responsibilities and full coverage. Provincial forest chiefs actively perform their duties by issuing forest chief orders, holding work deployment meetings, conducting forest patrols and conducting research, so as to accelerate the work of forest chief system in various places. In 2021, the provincial forest patrol was completed in full cooperation, and more than 270000 forest patrols were conducted by forest chiefs at all levels in the province.

[Vigorously developing under-forest economy] The CPC Guizhou Provincial Committee and the Guizhou Provincial People's Government issued the Opinions on Accelerating the High-quality Development of Under-forest Economy, and Guizhou Province became the first province to issue a document supporting the development of under-forest economy in the name of the Provincial Committee. The province's under-forest economic development area reached 1.8667 million hectares, the national under-forest economic demonstration base reached 30, the conversion rate of forest products processing reached 55.3%, the main body of implementation reached 17500, and the output value of the whole industrial chain reached 56 billion yuan. The "Implementation Plan for the Assessment of Accelerating the High-quality Development of Under-forest Economy in Cities and Counties" was promulgated, which was included in the assessment of "Consolidating and Expanding the Achievements of Poverty Alleviation and the Performance Evaluation Index of Rural Revitalization" in 2021. Complete the investigation and research project on major issues of the Provincial Party Committee, Statistical Monitoring and Research on the Development of Undergrowth Economy in Guizhou Province. To compile the Guidelines for the Utilization of Undergrowth Economic Forest Land with High Quality Development, and standardize the types, intensity and development modes of forest land utilization.

[Deep development of characteristic forestry industry] 194,700 hectares of characteristic forestry (bamboo, Camellia oleifera, Zanthoxylum bungeanum and Gleditsia sinensis) industrial bases have been newly built and cultivated, 17,300 hectares of walnut have been cultivated, and 23,300 hectares of fungus forest have been cultivated. The development scale of Gleditsia sinensis, Rosa roxburghii and Chimonobambusa quadrangularis industry ranks first in the country. Innovating the complementary and integrated development mode of "flower + tobacco" resources, building 10 pilot bases, the province's flower and seedling base reached 809000 hectares. To formulate normative standards such as Definition and Transformation Measures of Low-yielding Forests of Major Economic Tree Species in Guizhou Province (Trial Implementation) and Guidelines for Evaluation of Low-yielding Forest Transformation Projects in Guizhou Province (Trial Implementation). Organize a comprehensive survey of the current situation and development potential of resources in various regions, and grasp the base number of resources development. Guizhou characteristic forestry industry experience center and information release platform have been built, with more than 150 enterprises stationed in 27 categories and nearly 600 kinds of forest products. Promote the construction of Tongzi Bamboo Shoot Trading Center and put Zhijin Zaojiao Industrial Park into use.

[Forest quality improvement] Carry out pilot demonstration of tree species structure adjustment, and gradually adjust Chinese fir forest and Pinus massoniana forest in mature and over-mature commercial forests into high-value economic forest, precious forest or fungus timber forest through cutting and transformation measures, so as to improve the economic value and ecological value of forest. To compile and issue "Definition and Transformation Measures of Low-yielding Forest of Main Economic Forest Species in Guizhou Province (Trial)", "Technical Guidelines for Evaluation of Low-yielding Forest Transformation Projects in Guizhou Province (Trial)" and "Guidelines for Construction of Planting Bases of Main Characteristic Forest Species in Guizhou Province (Trial)" to provide technical support for forest management. To guide Shijingshan Forest Farm in Liping County and State-owned Forest Farm in Sandu County to complete the national forest management pilot task of 8 thousand hectares. The province has implemented a precise improvement of 197700 hectares of forest quality, including 40000 hectares of forest tending, 139600 hectares of low-yield and low-efficiency forest transformation, 14600 hectares of degraded forest restoration, 0.26 million hectares of timber strategic reserve base construction, and 0.09 million hectares of national special and rare forest cultivation. Two new national forest germplasm resource banks and four provincial forest germplasm resource banks have been built, and the general survey of forest germplasm resources in the province has been completed in an all-round way. Fourteen improved varieties of forest and grass were newly examined (approved).

China Forestry Yearbook, Guizhou Province, 2020

[Overview] In 2020, Guizhou Province completed 32.013 billion yuan of forestry investment, including 7.759 billion yuan of central financial funds, 3.906 billion yuan of provincial financial funds and 20.348 billion yuan of financing loans for national reserve forest projects. The province has completed 280000 hectares of afforestation, with the forest coverage rate reaching 61.51% and the forest stock volume reaching 609 million cubic meters; 131900 hectares of forestry characteristic industrial bases have been newly built and transformed, 1.4686 million hectares of under-forest economy have been developed, and the total output value of forestry has reached 337.8 billion yuan. The total number of ecological forest protection personnel has increased to 182,800, and 46,300 new forestry labor jobs have been created in the whole province; the compensation work for the withdrawal of artificially bred wild animals has been completed in an all-round way, the 4th China Greening Expo has been successfully held, and the Opinions on the Full Implementation of the Forest Chief System has been officially issued.

Forestry poverty alleviation will strive to add 7265 ecological forest rangers for the poverty-stricken population in Lika in 2020, 3000 provincial ecological forest rangers, and expand the scale of ecological forest rangers in the province to 182800. Through the development of ecological forest protection posts, the promotion of characteristic forestry industry, the development of under-forest economy, and the implementation of national reserve forest projects to promote employment, the province's forestry industry has provided a total of 46300 new jobs. We will increase support for Ceheng County, which is supported by the Forestry Bureau of Guizhou Province, and allocate 167 million yuan for forestry construction to help Ceheng County out of poverty.

Land greening carries out voluntary tree planting activities for provincial cadres. Through the implementation of key forestry projects such as returning farmland to forests, shelterbelts of the Yangtze River and the Pearl River, and the construction of characteristic forestry industrial bases, the province has completed 280000 hectares of afforestation and 382.26 ha of ecological restoration of degraded grasslands. We will complete the task of returning 218,600 hectares of farmland to forests in 2019 and strive for 199,900 hectares of farmland to forests by 2020. The credit area of the national reserve forest is 425600 hectares, the financing is 40.964 billion yuan, and the construction area is 134100 hectares. There are 975 million qualified seedlings, including 46.02 million Camellia oleifera, 10000 Zanthoxylum bungeanum 10595, 54.44 million bamboo, 45.44 million Chinese honey locust and 54.12 million Rosa roxburghii Tratt. The 4th China Greening Expo was successfully held in Duyun City.

The forestry industry has issued a special fund of 348.42 million yuan for the characteristic forestry industry. The total area of characteristic forestry base construction is 131,940 hectares, of which 70,200 hectares are newly built and 61,600 hectares are cultivated. The "Three-year Action Plan for the Development of Characteristic Forestry Industry in Guizhou Province (2020-2022)", "Guiding Opinions on the Development of Walnut Industry in Guizhou Province" and "Implementation Plan for Improving Quality and Efficiency of Walnut Cultivation in Guizhou Province (2021-2023)" were issued. Organize and participate in the third "Forest Food Exposition" in Shanghai, hold the docking meeting of investment attraction of Guizhou characteristic forestry (Beijing) in 2020 and the production and marketing of forest products of "Guizhou goods are popular all over the world", increase investment attraction, and sign 9 projects on the spot, with a total investment of 13.58 billion yuan. It has established cooperative relations with Zhonglin Group, Zhongmin State Control Group, Asia Agricultural Supply Chain Management (Beijing) Company, Shandong Heze Shangshan Water Company, Zhengda Group and other powerful enterprises. Build and operate Guizhou characteristic forest product experience center and information release platform, aggregate offline exhibition, publicity and display, on-site tasting, live broadcasting and other functions, 150 enterprises, more than 600 kinds of products.

Ecological protection has completed the investigation and assessment of the current situation and problems of nature reserves in the whole province, the formulation of rules for the integration and optimization of nature reserves at the local level, and the scientific evaluation of the protection value of various nature reserves at the local level. Jointly with the Department of Natural Resources of Guizhou Province, the Provincial Plan for the Integration and Optimization of Nature Reserves was submitted. Complete the task of recovering the forest vegetation restoration fee of "group communication" hardened roads in rural areas of the whole province, and complete the annual update of "one map" of forest resources management. We will continue to rectify outstanding problems such as the "six strict prohibitions" on forest protection, the "green shield" and the special action of law enforcement in nature reserves, especially the Chishui problem of the central environmental protection inspector. To promote the withdrawal and disposal of edible wildlife breeding, the province has verified that 1706 breeding entities have completed the withdrawal, 2.163 million animals on hand have been disposed of, and 176.7 million yuan of compensation funds have been cashed, with a cash rate of 100%. Steadfastly promoting the three-year campaign of special renovation of forest fire prevention and control safety, no major forest fires and casualties occurred in the province, and the rate of forest fire damage was 0.0059. We continued to do a good job in the prevention and control of forestry pests, and did a good job in the control of major forestry pests such as pine wood nematode disease. The task of control was overfulfilled, and the disaster rate of forestry pests was 0.167 ‰.

On September 17, the General Office of the CPC Guizhou Provincial Committee and the General Office of the Guizhou Provincial Government officially issued and implemented the Opinions on the Full Implementation of the Forest Chief System. The pilot work of optimizing and adjusting local public welfare forests and the pilot work of real estate registration of forest rights have been launched one after another. Guizhou Forest and Grass Development Co., Ltd. was established to increase capital and expand shares of Guizhou Forest International Travel Service Co., Ltd. to provide a provincial state-owned platform for the development of forestry economy in the province. Optimize the administrative approval process, jointly carry out the optimization of Guizhou power grid project approval services with Guizhou Power Grid Company and EHV Transmission Company, strengthen communication with Guizhou Transportation Department, Guizhou Water Conservancy Department and Guizhou Energy Bureau, actively promote the forward movement of service ports, further simplify the administration and decentralize power, complete the clearance of "one network operation" matters, and constantly optimize the business environment of Guizhou Province.

The basic guarantee promulgated "Some Opinions of Guizhou Forestry Bureau on Strengthening Scientific and Technological Work to Promote High-quality Development of Forestry (Trial Implementation)", and built a provincial forestry science and technology demonstration base of 5647 mu. More than 40 scientific researchers were selected from Guizhou Academy of Forestry Sciences to carry out counterpart assistance to state-owned forest farms. Establish various expert databases, such as grassland management experts, scenic spot evaluation experts, ancient and famous tree experts, construction project use forest land review experts, economic forest consultation experts, and establish forestry decision-making consultation expert committee to improve the level of scientific decision-making. Construction of Guizhou Ecological Vocational and Technical College started. Strengthen forestry propaganda work, publish "Typical Cases of Practicing 's Ecological Civilization Thought in Guizhou Province", launch 86 propaganda reports in national media, 457 propaganda reports on Guizhou forestry in media above provincial level, 31 special editions, and more than 3000 propaganda reports on Guizhou forestry in media above provincial level reproduced by major media. To organize the formulation of the 14th Five-Year Plan for Forestry Development.

[2020 provincial leaders' voluntary tree planting activity held] On March 12, the 2020 provincial leaders' voluntary tree planting activity was held in Guiyang. Liu Xiaokai, Deputy Secretary of the Provincial Party Committee, Chen Yiqin of the Provincial Governor and Chairman of the Provincial Political Consultative Conference, went to Xiaobi Village, Xiaobi Township, Shuanglong Airport Economic Zone, Guiyang City to carry out voluntary tree planting. Before the tree planting activities, Chen Yiqin and Liu Xiaokai watched the exhibition board of afforestation in Guizhou Province to understand the development of forestry and undergrowth economy in Guizhou Province. Deeply carrying out voluntary tree planting activities is an important measure to carry out 's ecological civilization thought and a concrete action to build a colorful Guizhou. The vast majority of cadres and masses in the province should thoroughly implement 's socialist ideology with Chinese characteristics in the new era and the important guiding spirit of General Secretary for Guizhou's work, cultivate the ecological civilization consciousness of the whole society, especially young people, adhere to the leadership of cadres and the whole people, regularly carry out afforestation activities, plant more trees and add more trees. Ensure that the forest coverage rate of the whole province will reach more than 60% by 2020, make Qianshan and Guishui more beautiful under the green decoration, and let the people of the whole province have more sense of gain and happiness in the ecological construction. Members of the Standing Committee of the Guizhou Provincial Committee, the Standing Committee of the Provincial People's Congress, the Provincial Government, the Leading Group of the Provincial Political Consultative Conference and the Party Group, the Provincial Military Region and the Guizhou Provincial Armed Police Corps are mainly responsible for comrades, and the President of the Provincial Court and the Procurator-General of the Provincial Procuratorate participate in the activities.

[The 4th China Greening Expo was held in Duyun] On October 18, the opening ceremony of the 4th China Greening Expo was held in Duyun City, Qiannan Prefecture. Liu Dongsheng, deputy director of the State Forestry and Grassland Administration, Hu Zhangcui, full-time deputy director of the National Greening Committee, Lan Shaomin, deputy secretary of the Guizhou Provincial Party Committee, Wu Qiang, Standing Committee of the Provincial Party Committee and Secretary General of the Provincial Party Committee, and Tao Changhai, deputy governor of the province. Luo Ning, vice chairman of the CPPCC Provincial Committee, and other leaders attended the opening ceremony. Lan Shaomin announced the opening ceremony, Liu Dongsheng and Tao Changhai made speeches respectively, and Hu Zhangcui presided over the opening ceremony. On November 18, the Green Expo closed smoothly. Focusing on the permanent theme of "People-oriented, Building a Green Home Together" and the sub-theme of "Green Circle China Dream, Hand in Hand into a Well-off Society", the Expo takes "New Picture Scroll of Green Water and Green Mountains, New Benchmark of Ecological Civilization" as its overall orientation, and "Guishan Guishui, Green Bo Qiannan" as its image orientation, with a total planning area of 1959 hectares, of which the core area is 399 hectares. It is the largest in all previous Green Expo Parks, with a total investment of about 3 billion yuan. The Green Expo has invited 57 units to build 56 outdoor exhibition gardens, and has held more than 1200 large and small events. During the period from the stress test on August 13 to the closing on November 18, 520,000 visitors visited the park. On December 9, Chen Yiqin, secretary of the Guizhou Provincial Party Committee and chairman of the Standing Committee of the Guizhou Provincial People's Congress, commented on the report of the Provincial Forestry Bureau on the holding of the Fourth China Greening Expo: The Green Expo was held very well. We should strengthen the operation and management of the Green Expo Park, give full play to its comprehensive benefits, and strive to make it an important window to show the achievements of the construction of ecological civilization in Guizhou.

On September 17, the General Office of the CPC Guizhou Provincial Committee and the General Office of the Guizhou Provincial People's Government issued the Opinions on the Full Implementation of the Forest Chief System, marking the preliminary completion of the provincial top-level design of the forest chief system in Guizhou Province and formally entering the stage of organization and implementation. The Opinions clearly States that the five-level forest chief system of provinces, cities, counties, townships and villages should be fully implemented throughout the province, and that the responsibility system with the Party and government leadership responsibility system as the core should be established and perfected. Party committees and governments at all levels are the main bodies responsible for the full implementation of the forest chief system, and forest chiefs at all levels are the first responsible persons for the protection and management of forest resources in the responsible areas. The joint meeting system of forest chiefs at the provincial, municipal, county and township levels has been established, which is responsible for studying and solving major problems in forest protection and development, formulating major decisions on forestry reform and development, setting up a linkage platform for departments, and defining new mechanisms such as regular tree planting activities for forest chiefs at the five levels, activities of "mountain patrol and forest protection" for forest chiefs at all levels, and the establishment of public signs for forest chiefs. It is clear that we should build a smart forest chief system, build a remote sensing data application platform for Guizhou forestry, and realize grid management of forest resources. It is necessary to establish a forest and grassland data monitoring platform with forest and grassland monitoring system as the main body, combine satellite remote sensing images and UAV technology, innovate the way of forest resources supervision and management, realize the dynamic monitoring and management of forest resources in Guizhou Province, and improve the innovation level of forestry digital management in Guizhou Province.

[Vigorously develop under-forest economy] The area of forest land used by under-forest economy in the whole province is 1.4686 million hectares, with an output value of 40 billion yuan, an increase of 21.2% over the same period last year. There are 17000 enterprises and professional cooperatives to develop the under-forest economy, which has increased the income of 2.85 million rural people. The area of forest land used for planting under forest is 212,000 hectares, including 8,400 hectares of edible fungi, 67,200 hectares of traditional Chinese medicinal materials and 136,100 hectares of others. The area of forest land used for breeding under forest is 223,800 hectares, including 25.6 million chickens, 619,000 boxes of bees and 649,000 livestock. The forest product collection and processing area is 464,400 hectares, including 7,533 tons of wild mushrooms, 5,907 tons of pine resin and 250,000 tons of bamboo shoots; the forest landscape utilization area is 568,300 hectares. In 2020, the comprehensive output value of ecotourism and forest recreation (including leisure services) will be about 187.5 billion yuan.

[Accelerating the development of characteristic forestry industry] In 2020, the province's characteristic forestry industry (bamboo, Camellia oleifera, Zanthoxylum bungeanum and Gleditsia sinensis) will be newly cultivated and cultivated for 131,900 hectares, of which 70,200 hectares will be newly cultivated and 61,600 hectares will be cultivated, with the output value of characteristic forestry reaching 16 billion yuan. The bamboo base covers an area of 316,600 hectares, of which the square bamboo area ranks first in the country. The processing of bamboo shoots in the province is 100,000 tons, with a processing rate of 40%; the processing rate of bamboo timber in the province is more than 80%. The Camellia oleifera base covers an area of 208,000 hectares, producing 80,000 tons of Camellia oleifera seeds and processing 44,000 tons of Camellia oleifera seeds in the province, with a processing rate of 55%, an increase of 17 percentage points over the same period of last year. Zanthoxylum bungeanum base covers an area of 79300 hectares, producing 53800 tons of fresh Zanthoxylum bungeanum, and the initial processing rate of fresh Zanthoxylum bungeanum is 100%. The Chinese honey locust base covers an area of 46,000 hectares, with the largest area of 20,000 hectares in Zhijin County, which is the largest distribution center for fine processing of Chinese honey locust in China. The processing rate of Gleditsia sinensis raw materials in the province is 100%. Li Zaiyong, member of the Standing Committee of Guizhou Provincial Party Committee, executive vice governor of Guizhou Provincial People's Government and leader of the Leading Group for the Development of Characteristic Forestry Industry, gave instructions on the progress of the work of characteristic forestry industry in 2020: In the past year, with the joint efforts of all of us, the development of characteristic forestry industry in Guizhou Province has achieved remarkable results. It has made an important contribution to winning the battle against poverty on time and with high quality. Entering a new stage of development, we should thoroughly implement the spirit of the Eighth Plenary Session of the Twelfth Session of the Provincial Party Committee, adhere to the overall situation of high-quality development, continue to give full play to our advantages, take advantage of the situation and overcome difficulties, and continue to strengthen the special forestry such as bamboo, Camellia oleifera, Zanthoxylum bungeanum and Gleditsia sinensis, so as to continuously turn Guizhou's green mountains into Jinshan and Yinshan.

[National Reserve Forest Construction] The Provincial Forestry Bureau, in conjunction with the State Development Bank of Guizhou Province, the Agricultural Development Bank of Guizhou Province and the Guizhou Financial Control Group, has actively explored and formed an integrated financing mode of "investment, loan and insurance" for the National Reserve Forest Project. "Investment" means that Guizhou Green Industry Poverty Alleviation Investment Fund supports the National Reserve Forest Project in the form of equity investment and plays the role of leveraging the Green Industry Fund. "Loan" means that financial institutions such as the Provincial State Development Bank and the Provincial Agricultural Development Bank support the national reserve forest projects in the form of medium and long-term loans, and play the main role of policy banks in financing. "Guarantee" refers to the way in which provincial financing guarantee companies provide guarantee and credit enhancement to bank loans, giving full play to the role of policy guarantee. By the end of 2020, the feasibility study of 192 projects in the province has passed the examination of industry experts, and 87 projects have been granted credit by banks, with a credit area of 420300 hectares and a total financing of 40.164 billion yuan. The completed construction area is 140,100 hectares, the implemented area ranks second in the country, and the amount of financing loans ranks first in the country. The construction of the national reserve forest project in the whole province has transferred 150500 hectares of forest land, 79700 hectares of forest land and 140100 hectares of implementation area, which has driven 204600 households and 715300 poor people to increase their income, with an average increase of about 18000 yuan per household. 15600 new jobs were created, exceeding the target of 15000 jobs set by the provincial government.

[Rectification of Chishui Problem of Central Environmental Protection Supervision] In view of the illegal use of forest land in Swan Castle and Tiandao Lake projects in Chishui City found by the Central Eco-environmental Protection Supervision Group, a rectification plan was formulated in time, and the Notice on Urging Chishui City to Implement the Rectification of Relevant Issues of Central Environmental Protection Supervision within a Time Limit was issued. Fourteen historical administrative cases have been punished, two criminal cases have been merged with later cases, and two cases have been filed and investigated by Chishui Public Security Bureau. In the two projects, the 23.86 hectares that can be restored to green in situ and the 9.11 hectares that need to be restored to green in other places have been fully completed. For the national public welfare forest occupied, according to the principle of "occupying one and compensating one", after consulting the State Forestry and Grass Administration and the Provincial People's Government for approval, Chishui City has been approved to supplement 101.02 hectares of public welfare forest. Accountability proposals have been put forward to the relevant responsible persons.

In order to implement the Decision of the Standing Committee of the National People's Congress on Prohibiting Illegal Wildlife Trade in an All-round Way, Abolishing the Bad Habits of Eating Wildlife Indiscriminately and Effectively Guaranteeing People's Life, Health and Safety, the Guizhou Provincial People's Government issued a circular and established Zhang Meijun, Director of the Provincial Forestry Bureau. Thirteen units, including the Department of Agriculture and Rural Areas of Guizhou Province, the Department of Finance of Guizhou Province and the Poverty Alleviation Office of Guizhou Province, are responsible for the inter-departmental joint meeting system for the withdrawal (conversion) of edible wildlife breeding in Guizhou Province. The joint meeting held two work promotion meetings successively, and supervised the withdrawal and conversion of edible wildlife breeding in 9 cities, prefectures, 16 counties, cities and districts of the province. As of December 31, the withdrawal and conversion of edible wild animals in the whole province has been completed in an all-round way, 1706 breeding entities have been verified to have been withdrawn, 2.163 million animals (articles) have been disposed of, and 176.7 million yuan of compensation funds have been cashed, with a cashing rate of 100%.

[Carry out the special action of forest supervision and "six strict prohibitions" law enforcement for forest protection] In combination with the forest supervision deployed by the State Forestry and Grassland Administration, the special action of "six strict prohibitions" law enforcement for forest protection in Guizhou Province will be carried out regularly. Completed the on-site investigation and verification of 20565 forest-related case clues, the verification rate of forest-related case clues was 100%; completed the administrative punishment of 3735 forest-related administrative cases, the investigation rate of forest-related administrative cases in the province in 2020 was 99.28%; In 2020, the transfer rate of 2087 forest-related cases that meet the criminal filing standards was 99.62%, and the fine collection and replanting of 2404 forest-related administrative cases in previous years were completed, with the penalty execution rate of 99.34%.

[Forestry Events]

On January 17, the Provincial Forestry Bureau issued a circular praising the outstanding collectives and individuals on the 20th anniversary of the construction of the project of returning farmland to forestry in Guizhou Province. Among them, there are 40 outstanding collectives and 199 outstanding individuals.

On February 1, the Regulations on the Protection of Ancient and Famous Trees in Guizhou Province came into effect.

On February 1, four local standards, namely, Guizhou Provincial Forest City Construction Standard, Guizhou Forest Township Construction Standard, Guizhou Forest Village Construction Standard and Guizhou Forest Home Construction Standard, were issued and implemented.

On February 17, 15 units, including the Provincial Health and Health Commission, the Provincial Development and Reform Commission, the Provincial Finance Department, the Provincial Education Department and the Provincial Forestry Bureau, jointly issued the Implementation Plan on Accelerating the Integration and Development of Medical and Health Services and Old-age Services, which included the compliance treatment projects of forest health care bases in the scope of medical insurance reimbursement, the first in the country.

On March 12, the voluntary tree planting activity of Guizhou provincial leaders was held in 2020. Liu Xiaokai, Deputy Secretary of the Provincial Party Committee, Governor Chen Yiqin, Chairman of the Provincial Political Consultative Conference, and others carried out voluntary tree planting in Xiaobi Village, Xiaobi Township, Shuanglong Airport Economic Zone, Guiyang City. Members of the Standing Committee of the Provincial Party Committee, the Standing Committee of the Provincial People's Congress, the Provincial Government, the Leading Group of the Provincial Political Consultative Conference and the Party Group, the Provincial Military Region and the Guizhou Provincial Armed Police Corps were mainly responsible for comrades, and the president of the Provincial Court and the chief procurator of the Provincial Procuratorate participated in.

On March 20, a teleconference on forestry work was held in Guizhou Province. The main venue was located in the provincial forestry Bureau, and the competent forestry departments and directly affiliated units of cities, counties, cities, districts and special zones set up branch venues respectively.

On April 2, the provincial government office issued a circular to establish an inter-office contact meeting system for the conversion (withdrawal) of edible wildlife breeding in the province.

On April 27, the National Development and Reform Commission and the State Forestry and Grass Administration announced the results of spot checks and acceptance of the reform of state-owned forest farms nationwide, and Guizhou Province entered the first phalanx with "excellent" results.

On May 12, a special study was made on the development of characteristic forestry, traditional Chinese medicine industry, ecological fishery and pepper industry. Li Zaiyong, Standing Committee of the Provincial Committee, Standing Vice Governor and Leader of the Provincial Leading Group for the Development of Characteristic Forestry Industry, Wang Shijie, Vice Governor and Leader of the Provincial Leading Group for the Development of Traditional Chinese Medicine Industry, Guo Ruimin, Vice Governor and Leader of the Provincial Leading Group for the Development of Eco-fishery Industry, and Wu Qiang, Vice Governor and Leader of the Provincial Leading Group for the Development of Pepper Industry, made reports and speeches respectively.

On June 4, the Forestry Bureau of Guizhou Province set up the Forest and Grassland Fire Prevention Department in accordance with the Reply on the Establishment of Internal Institutions by the Forestry Bureau of Guizhou Province (Qian Bian Ban Fa [2020] 77).

From June 5 to 7, Ding Zhongli, Vice Chairman of the Standing Committee of the National People's Congress, led the Law Enforcement Inspection Unit of the Standing Committee of the National People's Congress to inspect the Decision of the Standing Committee of the National People's Congress on the Comprehensive Prohibition of Illegal Wildlife Trade, the Abolition of Bad Habits of Eating Wildlife, and the Effective Protection of People's Lives, Health and Safety in Guizhou Province. Listen to relevant reports and hold discussions with NPC deputies, experts and scholars, and law enforcement personnel.

On June 17, with the consent of the Guizhou Provincial People's Government, the Guizhou Forestry Bureau issued the contingency plan for the Prevention and Control of Major Terrestrial Wildlife Epidemics in Guizhou Province, further strengthening the construction of the contingency plan system for the prevention and control of terrestrial wildlife epidemics in Guizhou Province, and providing a strong guarantee for the scientific disposal of major terrestrial wildlife epidemics.

On June 24, the Chen Yiqin of the Governor of Guizhou Provincial People's Government chaired a special meeting of the provincial government to study the assessment and adjustment of the red line of ecological protection and the integration and optimization of nature reserves in the whole province.

On June 24, Guo Ruimin, Vice Governor of Guizhou Provincial People's Government, Secretary of the Party Committee and Director of the Provincial Public Security Department, chaired a video conference on the implementation of the adjustment of the forest public security management system in the whole province, which made it clear that the forest public security in the whole province belonged to the local public security organs.

On September 17, the General Office of the Guizhou Provincial Committee and the General Office of the Guizhou Provincial People's Government issued the Opinions on the Full Implementation of the Forest Chief System.

On September 25, the 19th meeting of the Standing Committee of the 13th People's Congress of Guizhou Province decided to amend some provisions of the Regulations on Forest Land Management of Guizhou Province, in which the authority for examination and approval of temporary use of forest land was set up in an inverted pyramid at the provincial, municipal and county levels according to the type and area of forest land, and amended to "temporary use of forest land". "Temporary use of forest land in state-owned forest farms shall be examined and approved by the competent forestry authorities of the people's governments at the same level under the state-owned forest farms"; The examination and approval authority for the occupation of forest land by state-owned forest management units for the construction of engineering facilities directly serving forestry production within the scope of the forest land they operate shall be amended from "approved by the forestry administrative department of the provincial people's government" to "approved by the competent forestry department of the people's government at the same level under the state-owned forest farm", which guarantees the implementation of the requirement of "releasing control clothing" from the legal point of view.

On September 27, according to the "Approval on the Transfer of 112 Comrades in the Institutional Reform of the Forest Public Security Bureau of Guizhou Province", 93 special political and legal personnel and 112 personnel of the former Forest Public Security Bureau of Guizhou Province were transferred to the Public Security Department of Guizhou Province and the county (district) public security bureaus affiliated to the relevant municipalities and prefectures.

On October 18, the opening ceremony of the 4th China Greening Expo was held in Duyun City, Qiannan Prefecture. Liu Dongsheng, Deputy Director of the State Forestry and Grass Administration, Hu Zhangcui, Full-time Deputy Director of the National Greening Committee, Lan Shaomin, Deputy Secretary of the Guizhou Provincial Party Committee, Wu Qiang, Standing Committee of the Provincial Party Committee and Secretary-General of the Provincial Party Committee, Tao Changhai, Vice Governor, Luo Ning, Vice Chairman of the Provincial Political Consultative Conference, and other leaders attended the opening ceremony.

From October 18 to 19, Hu Zhangcui, full-time deputy director of the Office of the National Greening Committee, and his delegation went to Libo County to investigate forestry ecological poverty alleviation, and Zhang Fujie, deputy director of the Provincial Forestry Bureau, participated in the investigation.

From October 21 to 23, Li Chunliang, deputy director of the State Forestry and Grassland Administration, and his delegation conducted a survey on forestry poverty alleviation in Dushan and Libo counties of Guizhou Province. Li Tiansong, commissioner of Guiyang Commissioner's Office of the State Forestry and Grassland Administration, Zhang Meijun, director of the Provincial Forestry Bureau, and Xiang Shoudu, deputy director of the Provincial Forestry Bureau, participated in the survey.

On October 30, with the consent of the Guizhou Provincial People's Government, the Provincial Forestry Bureau issued the Opinions on Strengthening the Work of Forest and Grass Seedlings to Promote the High-quality Development of Forestry.

On November 16, the annual forest cutting quota compiled by Guizhou Province during the 14th Five-Year Plan period was approved by the State Forestry and Grassland Administration.

On November 18, the closing ceremony of the 4th China Greening Expo was held in Duyun City, Qiannan Prefecture. Hu Zhangcui, Full-time Deputy Director of the Office of the National Greening Committee, Huang Zhengqiu, Deputy Director of the Department of Ecological Protection and Restoration of the State Forestry and Grass Administration, Li Tiansong, Commissioner of the State Forestry and Grass Administration in Guiyang, Yang Bo, Deputy Director of the Publicity Center of the State Forestry and Grass Administration, He Gang, Deputy Secretary-General of the Guizhou Provincial People's Government, Zhang Meijun, Director of the Provincial Forestry Bureau, and Zhang Fujie, Deputy Director, attended the closing ceremony. Zhang Meijun presided over the closing ceremony and declared it closed.

On November 24, the National Labor Model and Advanced Workers Recognition Conference was held in Beijing. Twenty-four people in the national forestry and grass industry were commended, including yuan Changxuan, a staff member and senior engineer of the Forestry Industry Development Office of the Forestry Bureau of Tianzhu County, Guizhou Province, and Zhu Xin, an engineer and chief of the Personnel and Education Section of the State-owned Zazuo Forest Farm in Guizhou Province, who was awarded the title of National Advanced Worker.

On December 9, Chen Yiqin, secretary of the Guizhou Provincial Party Committee and chairman of the Standing Committee of the Guizhou Provincial People's Congress, commented on the report of the Provincial Forestry Bureau on the holding of the Fourth China Greening Expo: The Green Expo was held very well. We should strengthen the operation and management of the Green Expo Park, give full play to its comprehensive benefits, and strive to make it an important window to show the achievements of the construction of ecological civilization in Guizhou.

On December 18, the innovative practice of under-forest economy of Guizhou Forestry Bureau won the first prize of the target performance management innovation project of provincial organs in 2020.

On December 31, with the consent of the People's Government of Guizhou Province, the Standards for Collection and Use of Grassland Vegetation Restoration Fees in Guizhou Province and the Measures for Collection and Use of Grassland Vegetation Restoration Fees in Guizhou Province were promulgated and implemented on January 1, 2021.

China Forestry Yearbook, Guizhou Province, 2019

[Overview] In 2019, under the strong leadership of the CPC Guizhou Provincial Committee and the Guizhou Provincial People's Government, and with the strong support of the State Forestry and Grassland Administration, the forestry system of the whole province, guided by 's socialist ideology with Chinese characteristics in the new era, comprehensively implemented the important instructions of the Nineteenth National Congress of the CPC and General Secretary on Guizhou's work and forestry work. We should adhere to the overall situation of forestry work in order to fight against poverty, and closely combine the theme education of "never forget the original intention and remember the mission" with the tasks of promoting forestry reform and development, so as to promote the forestry work in the whole province to show a good development trend.

Land greening actively promoted the cultivation of forest seedlings, five new provincial-level forest germplasm resource banks were built, 23 large-scale breeding bases and nursery bases for characteristic germplasm resources were identified in the first batch, and 258 million seedlings (clusters) such as Dendrobium, Camellia oleifera, Rosa roxburghii, bamboo and Gleditsia sinensis were cultivated throughout the year, which led to 2.003 billion seedlings (clusters) in the whole province. We will implement key ecological projects such as afforestation subsidized by the central government, afforestation with vegetation restoration fees, comprehensive control of rocky desertification, and the construction of the "two rivers" shelter forest system, and strive for the task of returning farmland to forestry in Guizhou Province of 218700 hectares. Vigorously carry out social afforestation, the province's 186 thousand people participated in the five level cadres' voluntary tree planting, and the "Internet + national voluntary tree planting" continued to advance. Actively promote the greening and beautification of urban and rural environment, issue the Action Plan for Rural Greening and Beautification in Guizhou Province, the Development Plan for Forest Cities in Guizhou Province (2018-2025), and the Implementation Opinions on Accelerating the Construction of Forest City System, complete the survey and assessment of the greening coverage of 57 counties and villages in Guizhou Province, and promote the greening and beautification of urban and rural areas. Eleven provincial-level forest cities, 99 forest townships, 800 forest villages and 2800 forest households have been built, and 273 national forest villages in Guizhou Province have been identified by the State Forestry and Grass Administration. In the whole year, 5.2 million mu of afforestation was completed and 1006 square kilometers of rocky desertification were harnessed. Successfully completed the work of Guizhou Province in participating in the 2019 Beijing World Horticultural Exposition. Guizhou Indoor Exhibition Area won the first prize, Guizhou Outdoor Exhibition Garden won the gold prize, and Guizhou Forestry Bureau was awarded the "Best Organization Award" by the Organizing Committee of Beijing World Horticultural Exposition. In 2019, the Forest Tourism Festival was awarded "Best Organizational Unit" and "Best Provincial Exhibition Unit". In 2019, China Flower and Seedling Fair won the "Excellent Organizational Award" and "Best Creative Award". Actively organize the fourth Green Expo, and the Green Expo Park project is progressing in an orderly manner.

Ecological poverty alleviation completed the general survey of forest ecological industry resources. To promote the rural industrial revolution, the province added 102300 hectares of Dendrobium, Camellia oleifera, Rosa roxburghii and bamboo planting area, and transformed 57500 hectares of inefficient forests, with an output value of 18.24 billion yuan, which increased the income of 462500 poor people. Vigorously develop the under-forest economy, issue operational guidelines for the development of under-forest economy, formulate implementation plans focusing on the development of under-forest economy in deep poverty-stricken areas to promote poverty alleviation and fortification, hold on-site meetings on the development of under-forest economy and the construction of fungus timber forests in the whole province to promote poverty alleviation and fortification, build 42 demonstration projects of under-forest economy, and select cadres to carry out one-to-one projects. The economic area under forests in the province reached 1.3659 million hectares, with an output value of 22 billion yuan. There are 25500 new ecological forest rangers in the central finance, 87000 new ecological forest rangers for the poor in Lika, and 172500 ecological forest rangers for the poor in Lika in the whole province. The annual forestry investment was 29 billion yuan, and the central and provincial financial investment was 11.253 billion yuan, of which 9.198 billion yuan was invested in poverty-stricken counties, accounting for 81.74%, all of which reached a record high. Actively coordinate to raise the compensation standard for forest ecological benefits of local public welfare forests in the province from 10 yuan per mu to 12 yuan per mu, benefiting about 7.98 million people. To formulate and promulgate a number of measures to support Bijie pilot area to win the battle against poverty on time and with high quality, and invest 2.669 billion yuan in forestry construction in Bijie City. To carry out the work of helping Ceheng at designated points, 114 million yuan was allocated for forestry construction in Ceheng County throughout the year. Supporting the forestry construction funds of 198.08 million yuan in Dushan County and Libo County designated by the State Forestry and Grass Administration, including 91.55 million yuan in Libo County and 106.53 million yuan in Dushan County.

Resource protection actively carries out special actions such as national forest supervision, "Green Guard 2019" and "Six Strict Prohibitions" law enforcement for forest protection, and severely cracks down on all kinds of forestry and grassland violations. In 2019, the province investigated 4384 forestry administrative cases, fined 140 million yuan, replanted 568700 trees, and 4487 people were subject to administrative penalties. 1736 criminal cases involving forests were investigated and 1684 people were transferred to prosecution. We will vigorously promote the rectification of the central ecological environmental protection supervision and "looking back" feedback problems, and carry out the clean-up and rectification of illegal construction in protected areas. To sort out the basic situation of nature reserves in the whole province, and initially construct a "map" of nature reserves in the whole province. Establish the evaluation committee of local nature reserves in Guizhou Province, carry out the disposal of overlapping mining rights with nature reserves, and start the optimization and integration of nature reserves in the whole province. To draft the Opinions on the Implementation of the Natural Forest Protection and Restoration System in Guizhou Province (Draft), strengthen wetland protection and wildlife protection, increase the wetland protection rate to 49.65%, and the national key wildlife protection rate to 92%, and take the lead in completing the routine investigation task of "the second national survey of terrestrial wildlife resources". Actively promote the construction of forestry rule of law, promulgate and implement the Regulations on the Protection of Ancient and Famous Trees in Guizhou Province, promote the legislative work of the Regulations on the Prevention and Control of Forestry Pests in Guizhou Province, and carry out the revision of six laws and regulations such as the Regulations on the Management of Forest Land in Guizhou Province. Strict implementation of forest fire prevention and forest pest control responsibility, the annual fire damage rate of 0.0019 ‰, far below the national and Guizhou Province control indicators, forest pest disaster rate of 0.06 ‰, pine wood nematode and other major forest pest control rate of 100%.

Forestry reform comprehensively promotes the reform of forestry "release and control clothing", and jointly with Guizhou Power Grid Co., Ltd. and EHV Transmission Company, the implementation opinions on promoting the green development of Guizhou Power Grid are issued. A special class for handling forestry formalities for construction projects was set up to follow up relevant forestry formalities, 9975 forest land projects for construction projects were examined and approved throughout the year, and 2.169 billion yuan of forest vegetation restoration fees were collected. To vigorously promote the construction of national reserve forest projects and innovate forestry financing mode, the Construction Plan of National Reserve Forest Projects in Guizhou Province was approved by the State Forestry and Grass Administration, and the Opinions on Accelerating the Construction of National Reserve Forest Projects were issued by the General Office of the People's Government of Guizhou Province, striving for 732,000 hectares of national reserve forest construction tasks in the first phase, with a total investment of 60.4 billion yuan. Of the 77 planning and construction units, 53 feasibility study reports have passed the evaluation, 17 have been granted credit, with a total amount of 6.483 billion yuan, 2.875 billion yuan has been lent, and the National Reserve Forest Project has won the first prize of Guizhou Provincial Innovation Award. Opinions were drafted on the implementation of the reform of the forest chief system in an all-round way, and the pilot task of redeeming 15000 mu of artificial commercial forests in key ecological areas was completed, with 67 million yuan of redemption funds from farmers. Guizhou Provincial Party Committee's research project "Research on Guizhou Forestry Economic Index System" has passed the expert review, and the forestry statistics system has been approved and implemented by Guizhou Statistical Bureau, which is the first in the country. Jointly with the Guizhou Provincial Civil Affairs Department, the Guizhou Provincial Health and Health Commission and the Guizhou Provincial Administration of Traditional Chinese Medicine, it issued the Opinions on Promoting the Development of Forest Health Industry, which is the first one in China. The main task of state-owned forest farm reform has passed the on-site acceptance by the national acceptance team with excellent results. Promoting the reform of Jinping County, the collective forestry comprehensive reform pilot area of the State Forestry and Grass Administration, exploring the financing methods of forest right mortgage loan and forest right storage guarantee and the new forest management system, which was affirmed by the State Forestry and Grass Administration and the Guizhou Provincial Committee Reform Office, and Jinping County exchanged experiences at the national forestry comprehensive reform pilot area meeting.

Forestry scientific research has launched major special projects such as high-quality fungus timber forest, key technology research and industrial demonstration of Dendrobium candidum, 13 scientific research projects have been supported by the State Forestry and Grass Administration, 2 achievements have won the Guizhou Science and Technology Progress Award, and 1 has won the Liangxi Forestry Science and Technology Progress Award. Implementing 20 demonstration projects of transformation and standardization of scientific and technological achievements financed by the central government, building 11 provincial demonstration sites for forestry science and technology extension, building 4 national science and technology platforms such as the long-term scientific research base of the State Forestry and Grass Administration of Cunninghamia lanceolata and the National Innovation Alliance of Alpine Rhododendron, and establishing Guizhou Walnut Engineering Technology Research Center and Guizhou Branch of Dendrobium candidum Engineering Technology Research Center of the State Forestry and Grass Administration. The local standard system of Dendrobium, Rosa roxburghii and bamboo industries has been established and implemented. The construction of provincial forestry science and technology demonstration bases such as famous and excellent fruit forest demonstration bases has been promoted in an orderly manner. The National Forestry and Grass Science and Technology Poverty Alleviation Work Site Meeting was held in Libo, and the academic seminar commemorating the 60th anniversary of the founding of Guizhou Academy of Forestry Sciences was held pragmatically.

Self-construction cooperated with the completion of the fifth round of inspection team of Guizhou Provincial Party Committee to inspect Guizhou Forestry Bureau. The first batch of thematic education of the Bureau was approved by the tour guidance group of Guizhou Provincial Party Committee. The second batch of thematic education was successfully completed. There were 62 feedback problems from the inspection of Guizhou Provincial Party Committee, 57 problems have been rectified, 29 problems have been found in the inspection of thematic education, and 18 problems have been rectified. Some problems that have not been solved for a long time have been effectively solved, especially the relocation and comprehensive blockage of dangerous buildings in the East and West buildings of the Bureau compound, which have solved the problems that have not been solved for more than 10 years. Carry out work style rectification activities, set up work style rectification supervision group, and carry out work style inspection. The "July 1st" commendation conference of Guizhou Forestry Bureau was held to commend 12 advanced grass-roots Party organizations, 20 outstanding Communist Party members and 8 outstanding Party workers. Vigorously carry out a series of publicity work such as the 70th anniversary of the National Day and the 20th anniversary of the implementation of the project of returning farmland to forestry, and jointly carry out publicity activities on the theme of "keeping in mind the entrustment, keeping the bottom line, green water and green hills to see Guizhou" with a number of media in Guizhou Province. During the two sessions, the special edition of the Guizhou Provincial Political Consultative Conference reported the achievements of forestry ecological construction in Guizhou in 2019, and the media above the provincial level reported more than 1200 pieces of forestry information throughout the year. Successfully complete the institutional reform of the institutions directly under the Bureau, establish the cooperation mechanism between the departments of the Bureau and the institutions directly under the Bureau, and further enhance the vitality of forestry work.

On February 11, 2019, with the theme of "Building a Colorful Guizhou and Moving Towards a New Era of Ecological Civilization", the five-level synchronous voluntary tree planting activities in provinces, cities, counties and villages were held simultaneously throughout the province. Liu Xiaokai, deputy secretary of the Guizhou Provincial CPC Committee, Chen Yiqin of the provincial governor, and chairman of the Guizhou Provincial Committee of the Chinese People's Political Consultative Conference, participated in a tree planting activity at the Guizhou voluntary tree planting site in Fuguan Village, Guiyang Economic and Technological Development Zone. Since 2015, every year on the first day of work after the Spring Festival, we have organized five-level cadres in cities, counties and villages in Guizhou Province to carry out voluntary afforestation activities, forming a good tradition. This is an important measure taken by the Guizhou Provincial Party Committee and the Guizhou Provincial People's Government to implement the important instructions of General Secretary on keeping the two bottom lines of development and ecology, to implement the three strategic actions and to build the three national pilot zones. On that day, a total of 186000 people in the province participated in voluntary tree planting activities, with a total of 1.302 million trees planted.

On February 25, Chen Yiqin, Deputy Secretary of the CPC Guizhou Provincial Committee and governor of Guizhou Province, met with Ibrahim Tio, Executive Secretary of the United Nations Convention to Combat Desertification, who attended the Second Bureau Meeting of the Thirteenth Conference of the Parties to the United Nations Convention to Combat Desertification in Guiyang. Zhang Jianlong, Director-General of the State Forestry and Grassland Administration and President of the Thirteenth Conference of the Parties to the United Nations Convention to Combat Desertification, and other guests. Liu Jie and Wu Qiang, leaders of Guizhou Province, Pradeep Monga, Deputy Executive Secretary of the United Nations Convention to Combat Desertification, and Liu Dongsheng, deputy director of the State Forestry and Grassland Administration, attended the meeting. On February 26, the opening ceremony of the Second Bureau Meeting of the 13th Conference of the Parties to the United Nations Convention to Combat Desertification was held in Guiyang, with the main purpose of promoting global implementation and fully addressing the new challenges facing global desertification control. Zhang Jianlong, Director-General of the State Forestry and Grassland Administration and President of the 13th Conference of the Parties to the United Nations Convention to Combat Desertification, attended and delivered keynote speeches. Ibrahim Tiao, Executive Secretary of the United Nations Convention to Combat Desertification, Wu Qiang, Vice Governor of Guizhou Province, and Gou Haibo, Counsellor of the Ministry of Foreign Affairs, attended and delivered speeches. Liu Dongsheng, deputy director of the State Forestry and Grassland Administration, presided over the meeting. Pradeep Munga, Deputy Executive Secretary of the Convention, and country representatives of the United States, France and the Philippines attended the meeting.

[Guizhou Province participated in the 2019 China Beijing International Horticultural Exposition] From April 28 to October 9, Guizhou Province participated in the 2019 China Beijing International Horticultural Exposition. The Organizing Committee of Beijing World Horticultural Exposition awarded Guizhou Indoor Exhibition Area Special Prize and Guizhou Outdoor Exhibition Garden Gold Prize. Guizhou Forestry Bureau was awarded the Best Organization Award by the Organizing Committee of the Beijing World Horticultural Exposition. During the Expo, enterprises in Guizhou Province were organized to participate in various cut flower, potted plant, bonsai, flower arrangement and other competitions held by the Organizing Committee, and won 162 awards, including 17 special prizes, 38 gold prizes, 54 silver prizes and 53 bronze prizes, with a winning rate of 33.33% above the gold prize. The red kiwifruit selected by Liupanshui Liangdu Kiwifruit Industry Co., Ltd. won the gold medal in the high-quality fruit competition. During the Guizhou Provincial Day from August 4 to August 6, more than 20 events were held at the Expo. Wu Qiang, Vice Governor of Guizhou Province, attended the opening ceremony of the Guizhou Provincial Day and delivered a speech. Xu Zhihong, Chairman of the National Committee of the Chinese People and Biosphere Program, Chen Yong, Member of the Standing Committee of the Beijing Municipal Committee and Secretary of the Discipline Inspection Commission, Hu Zhangcui, Full-time Deputy Director of the Office of the National Greening Committee, and other leaders attended the opening ceremony. Chen Yiqin, the governor of Guizhou Province gave a "very good" comment on the participation of Guizhou Forestry Bureau.

[Carry out a general survey of forest ecological industry resources] In 2019, under the arrangement and deployment of the Guizhou Provincial People's Government, the provincial general survey of forest ecological industry resources was carried out. Guizhou province, city and county set up a three-level census leading group, set up a special work class, invited 21 experts to set up an expert committee, formulated corresponding technical standards, and identified 94 provincial technical instructors to conduct the whole process of technical guidance for the census work. The Report on the Results of the Census of Forest Eco-industrial Resources in Guizhou Province has passed the review jointly conducted by the State Forestry and Grass Administration and relevant experts in the province. This general survey comprehensively finds out the development of six major industries, namely, undergrowth planting, undergrowth breeding, characteristic economic forest, precious forest, forest tourism and forest health care, the suitable development area and the special resources of fungus timber forest and bamboo forest, so as to provide decision-making reference for the development of forestry characteristic industries in the province.

[Under-forest Economy] In 2019, Guizhou Forestry Bureau formulated and issued the "Implementation Plan for Focusing on the Development of Under-forest Economy in Deep Poverty-stricken Areas to Promote Poverty Alleviation" and "Guidelines for the Promotion of Under-forest Cultivation Projects in Extremely Poor Towns and Towns in Deep Poverty-stricken Counties of Guizhou Province", which included forest fungi, forest medicines, forest birds, forest bees, bamboo shoots and other short-term effective formats. Focusing on 16 poverty-stricken counties and 20 extremely poor townships in the province, we have allocated 64.58 million yuan to organize and implement 42 demonstration projects of under-forest economy, covering 18.8 million poor people. In 2019, the area of under-forest economic development in the province reached 853,100 hectares (excluding forest landscape utilization, the same below), an increase of 8.5% over the same period last year. The planting area under the forest has reached 237,300 hectares, the breeding area under the forest has reached 180,000 hectares, and a total of 23.24 million chickens, 501,500 boxes of bees and 956,700 livestock have been raised under the forest; the collection and processing area of forest products has reached 435,700 hectares. There are 14000 enterprises, professional cooperatives and other economic entities in the province to develop under-forest economy, which has increased the per capita income of 281000 poor people by nearly 1000 yuan.

[Forestry Characteristic Industries] On February 1, the General Office of the Guizhou Provincial Party Committee and the General Office of the Guizhou Provincial People's Government issued the Work System of Promoting the Rural Industrial Revolution under the Leadership of the Provincial Party Committee and the Provincial Government, defining 12 characteristic industries, such as Dendrobium, Camellia oleifera, Rosa roxburghii and bamboo, which were promoted by a Standing Committee of the Provincial Party Committee or a deputy governor. Guizhou Forestry Bureau, as the leading responsible unit for the development of Dendrobium and Camellia oleifera industries and the main responsible unit for the development of Rosa roxburghii and bamboo industries, has organized and formulated the implementation plan for the development of Dendrobium and Camellia oleifera industries, supported by key forestry projects, made every effort to build a high-quality industrial base, coordinated various forestry funds and provincial financial special funds of 1.116 billion yuan, and supported the whole industry chain. In 2019, the output value of the four major industries of Dendrobium, Camellia oleifera, Rosa roxburghii and bamboo was 18.24 billion yuan, driving 462500 people. Among them, the newly increased planting area of Dendrobium is 0.17 million hectares, the output of fresh Dendrobium is 7,207 tons, the output value is 3.548 billion yuan, and the income of poor people is increased by 47,100 people; the newly increased planting area of Camellia oleifera is 40,300 hectares, the cultivation area is 23,300 hectares, the output of Camellia oleifera seeds is 72,200 tons, the output value is 3 billion yuan, and the income of 109,000 people is increased; Bamboo planting area increased by 33,100 hectares, bamboo forests were cultivated by 21,000 hectares, bamboo timber was harvested by 684,700 tons, fresh bamboo shoots were produced by 200,000 tons, and the total output value was 8 billion yuan, which increased the income of 88,300 poor people. The planting area of Rosa roxburghii was increased by 27.2 million hectares, the cultivation area was 13.2 million hectares, the fresh fruit output was 67000 tons, the output value was 3.692 billion yuan, and the poverty-stricken population was 218.1 million.

[Fungus timber forest supply and protection] According to the raw material demand of 3 billion sticks for the development of edible fungi industry in Guizhou Province in 2019, the supporting construction of fungus timber forest base will strengthen the supply and protection of fungus timber in the province and build its own fungus timber base through the tending and thinning of existing forests, transformation and pruning and shaping of economic forests. The "Supply and Guarantee Scheme for the Development of Edible Fungi Industry in the Province in 2019", "Construction Plan for Fungi Forest Bases in Guizhou Province (2019-2022)", "Guiding Opinions on Supporting the Construction of Wood Fungi Bases and the Processing of Fungi Bases" were promulgated, and supporting policies such as key forestry projects supporting the construction of bases and subsidies for the production of fungi were adopted. To guide the main body of society to participate in the construction of fungus timber forest base and the production and supply of fungus timber. In 2019, a fund of 244 million yuan was implemented to build a fungus timber forest base of 33300 hectares with birch, oak and alder as the main tree species.

[Forestry Events]

On February 11, with the theme of "Building a Colorful Guizhou and Moving Towards a New Era of Ecological Civilization", the five-level synchronous voluntary tree planting activities in provinces, cities, counties and villages in 2019 were held simultaneously throughout the province. Liu Xiaokai, deputy secretary of the Guizhou Provincial CPC Committee, Chen Yiqin of the provincial governor, and chairman of the Guizhou Provincial Committee of the Chinese People's Political Consultative Conference, participated in a tree planting activity at the Guizhou voluntary tree planting site in Fuguan Village, Guiyang Economic and Technological Development Zone.

On February 15, the 2019 Provincial Forestry Work Conference was held in Guiyang. Wu Qiang, Vice Governor of Guizhou Provincial People's Government, attended the conference and made an important speech.

On February 19, the State Forestry and Grassland Administration announced the list of the first batch of national forestry and grassland long-term research bases, and the "National Long-term Research Base for Chinese Fir Breeding in Liping County" declared by Guizhou Academy of Forestry Sciences was approved to be established, which is the first national forestry and grassland long-term research base in Guizhou Province.

From February 25 to 28, the Second Bureau Meeting of the Thirteenth Conference of the Parties to the United Nations Convention to Combat Desertification was held in Guiyang City.

In March 12th, the launching ceremony of "tree planting e era every day 3.12"-2019 "e green Guizhou trip" Guizhou "Internet + national voluntary tree planting" was held in Guiyang Zhucheng square.

On March 22, Comrade Zhang Meijun was appointed Secretary of the Party Group of Guizhou Forestry Bureau by the Guizhou Provincial Committee.

On April 3, the People's Government of Guizhou Province held a teleconference on fire prevention in key areas such as forest in 2019 to arrange and deploy the work of forest fire prevention during the Qingming Dynasty. Li Zaiyong, Standing Committee of Guizhou Provincial Committee and Standing Vice Governor of Guizhou Province, made an important speech. Guo Ruimin, Vice Governor of Guizhou Province, presided over the meeting. Wu Qiang, Vice Governor of Guizhou Province, attended the meeting.

On April 10, Comrade Zhang Meijun was appointed Director of Guizhou Forestry Bureau by Guizhou Provincial People's Government.

On April 10, the Compilation Office of Guizhou Province issued the Reply on the Adjustment of the Organizational Establishment of Some Institutions under the Forestry Bureau of Guizhou Province, canceling the Forest Resources Management Station of Guizhou Province, and transferring the functions and approved establishment of the Station to the Natural Forest Protection Project Management Center of Guizhou Province; the Wetland Protection Center of Guizhou Province and the Public Welfare Forest Management Center of Guizhou Province were integrated to form the Wetland and Public Welfare Forest Protection Center of Guizhou Province; Guizhou Forestry Information Center was renamed as Guizhou Forestry Information and Publicity Center; Guizhou Forestry Foreign Cooperation Project Management Center was renamed as Guizhou Forestry Foreign Cooperation and Industrial Development Center.

On April 19, the Forestry Bureau of Guizhou Province issued the Notice on the Establishment of the First Guizhou Nature Reserve Assessment Committee to establish the Guizhou Nature Reserve Assessment Committee.

From April 28 to October 9, Guizhou Province participated in the 2019 China Beijing World Horticultural Exposition. The Organizing Committee of Beijing World Horticultural Exposition awarded Guizhou Indoor Exhibition Area Special Prize and Guizhou Outdoor Exhibition Garden Gold Prize. Guizhou Forestry Bureau was awarded the Best Organization Award by the Organizing Committee of the Beijing World Horticultural Exposition. During the Expo, enterprises in Guizhou Province participated in various cut flower, potted plant, bonsai, flower arrangement and other competitions organized by the Organizing Committee, and won 162 awards, including 17 special prizes, 38 gold prizes, 54 silver prizes and 53 bronze prizes. The red kiwifruit selected by Liupanshui Liangdu Kiwifruit Industry Co., Ltd. won the gold medal in the high-quality fruit competition. Wu Qiang, Vice Governor of Guizhou Provincial People's Government, attended the opening ceremony of Guizhou Provincial Day and delivered a speech. Xu Zhihong, Chairman of the National Committee of the Chinese People and Biosphere Program (ministerial level), Chen Yong, Standing Committee of the Beijing Municipal Committee and Secretary of the Discipline Inspection Commission, Hu Zhangcui, Full-time Deputy Director of the Office of the National Greening Committee, and other leaders attended the opening ceremony.

On May 15, the provincial Rosa roxburghii Tratt Industry Development Promotion Conference was held in Guizhou Provincial Government. Tao Changhai, Vice Governor of Guizhou Provincial People's Government, attended the meeting and made an important speech.

On May 15, the Work Program for the Special Action of "Six Strict Prohibitions" on Forest Protection in Guizhou Province in 2019 was issued by the General Office of the People's Government of Guizhou Province.

From June 8 to 9, the national theme publicity campaign of "Cultural and Natural Heritage Day" and "Green China Travel-Entering Beautiful Tongren Large-scale Thematic Public Welfare Activities" were held in Tongren City. Li Chunliang, Member and Deputy Director of the Party Group of the State Forestry and Grassland Administration, Chen Jian, Vice-Chairman of the Guizhou Provincial Political Consultative Conference, Zhang Xinsheng, Chairman of the Council of the World Conservation Union, Liu Jiaqi, Academician of the Chinese Academy of Sciences and Famous Geologist, Zhou Jiagui, Deputy Secretary-General of the Secretariat of the National Committee of UNESCO of China, More than 500 leading guests, including Zhang Meijun, Director of Guizhou Forestry Bureau, and Miao Jie, Deputy Director of Guizhou Forestry Bureau, attended the event.

On July 5, the People's Government of Guizhou Province set up a special leading group for the rectification of nature reserves in Guizhou Province, with Lu Yongzheng, Vice Governor of the People's Government of Guizhou Province, as the group leader, Tao Changhai and Wu Qiang, Vice Governors of the People's Government of Guizhou Province, as the deputy group leaders, and comrades in charge of relevant departments, bureaus and municipalities and prefectures as members, with an office in the Forestry Bureau of Guizhou Province.

On July 10, with the consent of the People's Government of Guizhou Province, the Forestry Bureau of Guizhou Province, the Civil Affairs Department of Guizhou Province, the Health Commission of Guizhou Province and the Administration of Traditional Chinese Medicine of Guizhou Province jointly issued the Opinions on Promoting the Development of Forest Health Industry.

On July 19, the Notice on the Comprehensive Investigation and Assessment of Nature Reserves was issued by the General Office of the People's Government of Guizhou Province.

On July 30, the Opinions of the General Office of the People's Government of Guizhou Province on Accelerating the Construction of National Reserve Forest Projects were issued and implemented by the General Office of the People's Government of Guizhou Province.

On August 7, the Forestry Bureau of Guizhou Province issued the Guidelines for the Promotion of Undergrowth Cultivation Projects in Extremely Poor Towns and Villages in Deep Poverty-stricken Counties of Guizhou Province.

On September 18, the Guizhou Provincial People's Government signed a strategic cooperation framework agreement with the International Bamboo and Rattan Center.

On October 16, Guizhou Forestry Bureau, Guizhou Power Grid Co., Ltd. and Guizhou Super High Transmission Company jointly issued the Notice on Optimizing Services to Promote the Green Development of Guizhou Power Grid, and introduced specific measures on how to further improve the protection of forest and grassland resources and power grid construction, operation and maintenance, and power transmission from west to east.

On October 24, Wu Qiang, Vice Governor of Guizhou Provincial People's Government, chaired a special meeting of the Leading Group on Natural Forest Resources Protection and the Project of Returning Farmland to Forestry, and arranged for the deployment of related projects in 2019.

On October 25, the feedback meeting on on-site acceptance of state-owned forest farm reform was held in Guiyang. The fifth national acceptance team reported the on-site acceptance results, and the on-site acceptance results of the reform of state-owned forest farms in Guizhou Province were "excellent", and the evaluation of "three pioneers" was given, that is, the reform of state-owned forest farms in Guizhou Province took the lead in formulating local laws and regulations, taking the lead in defining all state-owned forest farms in the province as public welfare institutions, and taking the lead in defining the acceptance scores of each forest farm in the provincial commitment letter.

From October 27 to 30, the Fourth National Symposium on Chinese Fir and the Conference on Chinese Fir Industry Promoting Poverty Alleviation was held in Liping County, Qiandongnan Prefecture, sponsored by the Chinese Forestry Society and co-sponsored by the Guizhou Academy of Forestry Sciences. More than 200 Chinese fir experts and scholars from more than 40 units across the country participated in the conference.

From November 5 to 8, Peng Youdong, deputy director of the State Forestry and Grass Administration, and his delegation went to Libo, Dushan and Guiyang in Guizhou Province for investigation, during which they attended the National Forestry and Grass Science and Technology Poverty Alleviation Site Meeting held in Libo and made an important speech at the meeting.

On November 18, the Guizhou Provincial People's Government approved the establishment of Guizhou Eco-Vocational and Technical College for a period of 1-2 years.

On November 27, the Forestry Bureau of Guizhou Province issued the Implementation Plan on Focusing on the Development of Under-forest Economy in Deep Poverty-stricken Areas to Promote Poverty Alleviation.

On November 28, the Forestry Bureau of Guizhou Province issued the Implementation Opinions of the Forestry Bureau of Guizhou Province on Further Liberalizing the Rural Collective Forest Land Management Rights, which further liberalized the rural collective forest land management rights and improved the policy system of the rural collective forest land management rights.

On November 29, the Leading Group of the Special Work on the Rectification of Nature Reserves in Guizhou Province issued the Notice of the Leading Group of the Special Work on the Rectification of Nature Reserves in Guizhou Province on the Optimization and Adjustment of Nature Reserves in Guizhou Province. The rules of "Three Districts to Two Districts" and the Rules of Local Nature Reserve Scope Adjustment (Trial Implementation) and the Rules of Integration and Merger of Nature Reserves in Guizhou Province (Trial Implementation) were issued, which marked the full start of the optimization and adjustment of nature reserves in Guizhou Province.

On December 1, the Regulations on the Protection of Ancient and Famous Trees in Guizhou Province were adopted by the Standing Committee of the Thirteenth People's Congress of Guizhou Province at its thirteenth meeting and came into effect on February 1, 2020.

On December 2, the Forestry Bureau of Guizhou Province issued the Guiding Opinions on Supporting the Construction of Wood Fungus Base and the Processing of Fungus, encouraging all kinds of social subjects to participate in the construction of Fungus Forest Base and the processing of Fungus, effectively guaranteeing the supply of raw materials for Fungus and promoting the development of edible fungus industry.

On 4 December, Guizhou Provincial Market Supervision Bureau approved and issued Guizhou Provincial Forest City Construction Standard (DB52/T1455-2019), Guizhou Forest Township Construction Standard (DB52/T1456-2019), Guizhou Forest Village Construction Standard (DB52/T1457-2019), The Standard for Forest Home Construction in Guizhou Province (DB52/T1458-2019) is a local standard.

On December 5, Guizhou Rural Industrial Revolution Bamboo Industry Development Docking Meeting opened in Chishui City. With the theme of "Integration, Sharing, Innovation and Development", Lu Yongzheng, Vice Governor of Guizhou Provincial People's Government, attended the meeting and made an important speech. At the meeting, the establishment of Guizhou Bamboo Industry Consortium was announced.

On December 6, two new technologies invented by Lan Hongbo and others in Guizhou Maolan National Nature Reserve, including "a Soil Animal Sample Collection Box" (Patent No.: ZL 201920308723.6) and "Portable Small and Medium-sized Soil Animal Separation Device (Patent No.: ZL201920308144.1)", were granted national utility model patents.

On December 10, the Office of Guizhou Greening Committee and Guizhou Forestry Bureau awarded 11 counties (cities and districts) the title of "Forest City of Guizhou Province", 99 townships (towns) the title of "Forest Township of Guizhou Province", 800 villages the title of "Forest Village of Guizhou Province" and 2800 households the title of "Forest Family of Guizhou Province".

On December 17, 132 villages in China were awarded the title of "National Eco-cultural Village" after the preliminary and second evaluation by experts organized by the China Eco-cultural Association. Six villages in Guizhou Province were awarded the title of "National Eco-cultural Village", which is one of the provinces and regions that have been awarded the title of "National Eco-cultural Village" this year.

On December 19, Guizhou Forestry Bureau led the major project of Guizhou Provincial Committee in 2019, Guizhou Forestry Statistics Research, which passed the acceptance of experts organized by the Policy Research Office of Guizhou Provincial Committee.

On December 25, the State Forestry and Grassland Administration recently issued the Notice on the Acceptance of Pilot National Wetland Parks in 2019, and 10 pilot national wetland parks in Guizhou Province passed the acceptance.

In the second survey of wildlife resources, which began in December 2011, Guizhou Province completed the survey of six geographical units in Guizhou, becoming the earliest province in the country to complete this work.

In December, the State Forestry and Grass Administration identified 273 villages in Guizhou Province as national forest villages.
