# Supplementary material for: Exploration of forestry carbon sequestration practice path in Guizhou province-based on evolutionary game model
Source: PLoS One. 2024 Dec 13;19(12):e0314805. doi: 10.1371/journal.pone.0314805 (PMC11643302; doi:10.1371/journal.pone.0314805)
Supplement: Attachment — Submitted filename: Review Comments Tr.docx [file pone.0314805.s002.docx]

**General Assessment:**

- **Technical Rigour**: The study presents a clear, well-structured approach to evaluating the forestry carbon sequestration practice path using the evolutionary game model. However, there are areas where clarification or expansion of specific sections would enhance the manuscript's rigor.
- **Clarity and Coherence**: While the manuscript is generally well-written, there are several areas where language improvements or clarifications are necessary. These would improve readability for non-specialists and ensure better communication of key points.

**Specific Comments:**

**Abstract**

1. **Page 1, Lines 12-26**:
   - **Clarity**: The abstract is somewhat dense and could benefit from clearer language. For instance, the sentence, *"the ESG score and the amount of government subsidies have a significant positive impact on the amount of forestry carbon sequestration purchased by enterprises..."* could be simplified for a broader audience.
   - **Recommendation**: Consider breaking down complex ideas into simpler statements and possibly reducing jargon.

**Introduction**

2. **Page 2, Lines 33-91**:
   - **Context**: The introduction effectively outlines the importance of China's "double carbon" goals and sets the stage for the research. However, there is a lack of international context. How do China's efforts compare to other countries?
   - **Recommendation**: Briefly mention global efforts in forestry carbon sequestration to provide international context.
3. **Page 2, Line 86-91**:
   - **Linkage**: While the introduction covers the macro and micro aspects of carbon sequestration and ESG, the linkage between these two levels can be made clearer.
   - **Recommendation**: Explicitly outline how the macro and micro designs combine to create the proposed mechanism.

**Literature Review**

4. **Page 3-4, Lines 92-205**:
   - **Critical Review**: The literature review is comprehensive, but it could benefit from more critical analysis of the gaps in previous research, particularly regarding the shortcomings of ESG systems globally.
   - **Recommendation**: Strengthen the critique of past research to better justify the need for the current study.

**Methodology**

5. **Page 6, Lines 207-248**:
   - **Game Model Clarification**: The operation mechanism and evolutionary game model are central to the paper, but the explanation is overly complex and technical. Simplification and additional explanation would benefit readers unfamiliar with game theory.
   - **Recommendation**: Provide a short primer on evolutionary game theory to help readers who may not be familiar with it. Adding a diagram could also aid understanding.
6. **Page 9, Line 407**:
   - **Weighting Criteria**: The paper uses the Analytic Hierarchy Process (AHP) to determine weights in the ESG rating system. The AHP process is mentioned, but further details on how experts were chosen and how scores were aggregated are needed.
   - **Recommendation**: Provide more detail on the AHP process, including how experts were selected and how subjectivity was minimized.

**Results**

7. **Page 14, Lines 572-602**:
   - **Descriptive Statistics**: The results section is clear, but the descriptive statistics could benefit from more discussion. How do these statistics compare with existing data or studies in this field?
   - **Recommendation**: Compare the descriptive statistics with similar studies to highlight the uniqueness of your findings.

**Discussion**

8. **Page 15, Lines 626-655**:
   - **Policy Implications**: The discussion mentions policy implications, but they are somewhat general.
   - **Recommendation**: Be more specific about the implications of your findings for policymakers, particularly in terms of forestry carbon sequestration policies in China and abroad.
9. **Page 15-16, Lines 633-641**:
   - **Industry Comparison**: The heterogeneity analysis across industries is a strong point of the paper. However, more detail is needed on why certain industries (e.g., chemical or textile industries) are affected differently.
   - **Recommendation**: Expand on the reasons behind the varying effects of ESG and government subsidies across different industries.

**Writing and Organization**

10. **General Writing**:
    - **Language**: There are several instances where the language is overly technical or complex.
    - **Recommendation**: Simplify language and sentence structure throughout the manuscript for broader accessibility.
11. **Structure**: The manuscript is well-structured but would benefit from clearer section transitions.
    - **Recommendation**: Use more explicit headings or sub-headings to break up dense sections, particularly in the methodology and results.

In general, the study provides important insights into forestry carbon sequestration in China and offers a novel approach using an evolutionary game model. However, improvements can be made in terms of clarity, depth of analysis, and the presentation of results.
