# Supplementary material for: Exploration of forestry carbon sequestration practice path in Guizhou province-based on evolutionary game model
Source: PLoS One. 2024 Dec 13;19(12):e0314805. doi: 10.1371/journal.pone.0314805 (PMC11643302; doi:10.1371/journal.pone.0314805)
Supplement: Attachment — Submitted filename: Response to Reviewers10.25.doc [file pone.0314805.s003.doc]

Dear editor：

Thank you very much for your letter. We have learned much from your and seven reviewers’ comments, which are fair, encouraging and constructive. After carefully studying the comments and your advice, we have made corresponding changes. The main revisions are listed below.

# For reviewer 1:

## First of all, thank you very much for your and the other six reviewers' suggestions. These suggestions have benefited us a lot and brought a lot of quality improvement to the article. In response to your question, I have made the following changes

1- The novelty and methodology, must be presented carefully and clearly in the abstract
2-The abstract section needs to be clearer and more effective.‎ It is suggested to be re-written summarizing a short introduction, problem statement, ‎methodology, major results, and conclusion and recommendation
3- Implication of study results, conclusion, and recommendation for future must be well defined in the abstract
 **Thank you for the reviewer's comments. We have also found many issues with the abstract, including a lack of methodological presentation and novelty. Based on your three suggestions, we have rewritten the abstract：**

Guizhou Province has abundant forest resources, and it has great economic value and social benefits to explore the practical path of forestry carbon sequestration. Based on the current situation of forestry carbon sequestration development in Guizhou Province, this paper innovatively integrates forestry carbon sequestration indicators into the existing Environmental, Social and Governance(ESG) evaluation system using an evolutionary game model. It analyzes the factors restricting forestry carbon sequestration and explores the influencing factors of forestry carbon sequestration benefit sharing bodies in Guizhou. Through regression analysis, the paper discusses the impact of enterprise ESG scores, government subsidy amounts, and forestry carbon sequestration costs on forestry carbon sequestration purchase volume. The research results show that enterprise ESG scores and government subsidy amounts have a significant positive impact on enterprise forestry carbon sequestration purchase volume, while forestry carbon sequestration costs have a significant negative impact. The results have passed the robustness test in different industries. The simulation analysis results show that the stable point of the evolutionary game is (1,0,1) and (1,1,0), which verifies that the ESG rating system with forestry carbon sequestration integration can promote enterprises to purchase more forestry carbon sequestration, i.e., the effectiveness of forestry carbon sequestration in activating the ESG rating system mechanism. Based on the research conclusions, the paper puts forward policy implications: the government should accelerate the construction of localized ESG rating systems, improve enterprise information disclosure and supervision, increase subsidies and reduce forestry carbon sequestration costs, and optimize carbon quota design.

4.The abbreviations as such CCER and ESG must put in full name in in the abstract and throughout the manuscript

#  Thanks to the opinions of the reviewer, We have made corresponding modifications,We used the full name when the professional name first appeared, followed by an abbreviation.

4- Introduction: Carbon enterprises, needs to be introduced in more detail, as its types, distribution in China and globally and impacts on environmental and public health over the last years
5- What are the strategies and technologies used to control the Carbon enterprises?
6- The advantages and disadvantages of building of carbon market over recent years
What criteria were used to select the control technologies?
 . Thanks to the opinions of the reviewer, .We have made corresponding modifications,We have added these contents in the introduction section

Introduction

Since the reform and opening up, the Chinese economy has experienced rapid growth. Energy consumption has provided support for economic growth, but it has also resulted in significant carbon emissions, leading to environmental damage caused by excessive greenhouse gas emissions. China's economic growth and carbon emissions are in a "weak decoupling" state, which means that there may be outdated emission reduction technologies and ineffective emission reduction management methods in China's energy consumption. With the signing of the Paris Agreement and China's increasing carbon emissions, China is facing heavier international pressure to reduce greenhouse gas emissions. As a "responsible major power," China has been making its own efforts and contributions to addressing climate change, actively exploring and trying to establish a carbon emissions trading market to promote the low-carbon emission of high-emission enterprises and suppress the continued rise of domestic carbon emissions using market means[1-5].(Figure 1)

Figure 1.China's Energy and Climate Policy Plan

In order to scientifically reduce the emissions of high-carbon enterprises, China has designed from the macro and micro levels. At the macro level, the regulatory authorities will include high-carbon enterprises in the scope of emission control, through accelerating the construction of carbon market, building a scientific and orderly carbon trading system, with the help of market mechanism to achieve carbon emission reduction[6-10]. In the process of improving the construction of carbon market, China Certified Emission Reduction (CCER) project trading market is an important way to reduce emissions in the carbon market. It was officially restarted under the document "Measures for the Management of Voluntary Greenhouse Gas Emission Reduction Trading (Trial Implementation)" issued by the Ministry of Ecology and Environment in 2023. As an indispensable carbon offset product in the CCER market, forestry carbon sequestration mainly uses forests to absorb and fix carbon dioxide through afforestation, forest management and other activities, which has significant advantages in ecological benefits, is an important innovative way to achieve the goal of carbon neutrality, and faces new development opportunities. However, in the actual CCER market, the purchase demand of enterprises for forestry carbon sinks is insufficient, and the development of forestry carbon sinks is restricted. At the micro level, the regulatory authorities urge high-carbon enterprises to transform and increase efficiency through mandatory disclosure of environmental information. Among them, ESG (Environmental, Social and Governance) is a new investment concept and evaluation tool in recent years, covering the three dimensions of environmental, social and corporate governance information. With the help of ESG information disclosure and rating system, investors can evaluate the comprehensive operation and sustainable development ability of an enterprise in a multi-dimensional and all-round way, and then influence the decision-making of the enterprise. Qiu[11]proposed that good ESG performance can ease the financing constraints of enterprises; Li[12] believed that a complete ESG rating system is an important starting point to achieve the "double carbon" goal; Hu[13]emphasized that ESG rating can significantly promote the green transformation of enterprises through market incentives and external supervision mechanisms. To sum up, ESG has the function of reducing financing costs and enhancing enterprise value, which is of great significance to promote emission reduction of high-carbon enterprises.

The above macro-level and micro-level designs are effective ways to promote emission reduction of high-carbon enterprises, but they often play an independent role and do not establish an effective linkage mechanism. As far as the carbon market mechanism is concerned, due to the sufficient supply of market quotas and the relative price advantage of excess carbon quotas, emission control enterprises usually tend to choose to purchase excess quotas to offset excess emissions, rather than forestry carbon sequestration projects to achieve carbon sequestration at the ecological level. Therefore, how to promote high-carbon enterprises to purchase forestry carbon sinks spontaneously from the market mechanism is of great significance to the realization of the goal of carbon neutrality. In order to solve the problem of insufficient demand for forestry carbon sinks, ESG may become an innovative way to promote emission reduction of high-carbon enterprises at the micro level. Qian [14]pointed out that ESG has the ability to guide the flow of funds to green low-carbon areas. It can be seen that an effective ESG mechanism can guide high-carbon enterprises to buy more forestry carbon sinks.

According to China's dual carbon policy, carbon emitting enterprises face rigorous postgraduate entrance examinations. By 2060, as coal-fired power plants and coal based industrial processes that have not adopted emission reduction measures have been basically eliminated, the proportion of coal combustion related emissions will be reduced by about 50% compared to 2020. During the period of 2021-2060, process emissions (inherent emissions generated by chemical reactions in industrial processes) will decrease by about 90%, and the proportion of total emissions will almost double, due to the fact that it is extremely difficult to eliminate process emissions in certain heavy industry sectors, especially the cement and steel industries. The remaining emissions of the energy system by 2060 will be fully offset by negative emissions generated by BECCS and direct air capture and storage. In China's efforts to achieve full economic greenhouse gas neutrality before 2060, carbon removal technology can also be used to offset some of the more difficult to reduce non carbon dioxide greenhouse gases. Therefore, the carbon sequestration capacity of ecosystems, especially forestry carbon sequestration, is particularly important.(Figure 2)

Guizhou Province is located in western China and has state-owned forest areas, ranking high among all provinces in terms of forest area. After the implementation of the comprehensive ban on logging natural forests in state-owned forest areas, the accumulation area and quality of forests in Guizhou Province have been significantly improved, providing a unique natural resource advantage for the development of forestry carbon sinks in Guizhou Province. However, according to the data from China's voluntary emission reduction trading information platform, the development potential of forestry carbon sinks in Guizhou Province has not been fully activated, and the supply of forestry carbon sinks is relatively insufficient[14-18]. As of 2023, the implementers of forestry carbon sequestration projects in Guizhou Province are all local forestry bureaus. This indicates that the supply subject of forestry carbon sink in Guizhou Province is relatively single. Forestry carbon sequestration projects have the characteristics of large initial investment, long cycle, and difficulty as collateral for mortgage loans. The single supply subject of forestry carbon sink will increasingly constrain the development of forestry carbon sink in Guizhou Province, affecting the stability of effective supply of forestry carbon sink in Guizhou Province, and thus affecting the realization of ecological and social benefits of forestry carbon sink. Compared to Guangdong Province's carbon inclusive mechanism and other forestry carbon sink development policies, Guizhou Province currently does not have a systematic forestry carbon sink development policy and support system. This hinders the improvement of the external environment for the development of forestry carbon sinks in Guizhou Province, affects the stability of economic benefits that forestry carbon sink suppliers can obtain, and is not conducive to improving the enthusiasm of forestry operators to provide forestry carbon sinks, thereby affecting the effectiveness and stability of forestry carbon sink supply in Guizhou Province, leading to a vicious cycle in which the ecological and social benefits of forestry carbon sinks are difficult to achieve. When forestry management enterprises and governments form a forestry carbon sink benefit sharing body, that is, when a "cooperative win-win" model of forestry carbon sink is formed, it can effectively improve the stability of effective supply of forestry carbon sink, achieve the ideal cycle of ecological, social and economic benefits of forestry carbon sink, and promote the sustainable development of forestry carbon sink in Guizhou Province.[19-24]

In summary, this paper takes the current development status of forestry carbon sinks in Guizhou Province as the starting point, analyzes the behavioral characteristics of stakeholders in forestry carbon sinks in Guizhou Province, constructs an evolutionary game model to analyze the influencing factors of the construction of forestry carbon sink benefit sharing bodies in Guizhou Province, and proposes countermeasures and suggestions to promote the stable and innovative development of forestry carbon sinks in Guizhou Province based on the analysis results. On the one hand, this can fully and effectively utilize the forest resources in Guizhou Province to promote the industrial and orderly development of forestry carbon sinks, accelerate the speed of China's greenhouse gas emissions reduction, and contribute to the development of the national ecological economy. On the other hand, this can broaden the ways of ecological civilization construction in Guizhou Province, provide new economic development channels for state-owned forest areas, and promote the sustainable development of forestry carbon sinks in Guizhou Province

7- How to validity of the evolutionary game model? How was the data from different sources synthesized and analyzed to ensure consistency and reliability?
   8-What are the key gaps in the current understanding of ESG impact on socioeconomic and ecosystems? How do these gaps inform future research directions and policy development?
   8- The study identifies several areas for future research, such as the need for more stringent monitoring and tailored source control strategies.
   What specific methodologies or technologies hold the most promise for advancing the understanding and management of ESG ? How can these be incorporated into future research efforts?
   9- How does this study build upon or differ from previous studies on their impacts? What new insights or perspectives does it offer?
   10.What criteria were used to select the studies included in this model? How were these criteria applied to ensure the inclusion of relevant and high-quality application?
   11.How was the data from this model synthesized and analyzed? What methods were used to ensure consistency and reliability in the findings?
   . Thanks to the opinions of the reviewer, This series of questions can be summarized as how to determine the reliability and effectiveness of the current model. In response to the above issues, we have supplemented the article by adding the following chapters:

2.3. The Development of Evolutionary Game Theory and Model Application Cases

5.3. Model validation

(6)Establish a sound forestry carbon trading market in Guizhou Province

12.What are the key gaps in the current study? How do these gaps inform the recommendations for future research and policy development?
13.How could the structure to be improved to enhance readability and accessibility for a broader audience?
14- More figures and tables must be added to strengthen and eases the article contents by the readers
15- Limitations of this study must state clearly
16- Conclusion must be customized stating the main results and the significant impact of this study on economics, society and the recommendation for future
17- The reference. The author needs to provide more updated references, especially in the introduction and discussion.

. Thanks to the opinions of the reviewer, We have removed some redundant explanations and added some images to facilitate readers' understanding. For the added parts, we have also supplemented the references. The reviewers have provided many good suggestions. Thank you again.We have made corresponding modifications

# For reviewer 2:

## First of all, thank you very much for your and the other six reviewers' suggestions. These suggestions have benefited us a lot and brought a lot of quality improvement to the article. In response to your question, I have made the following changes

1- . Generally, it is better to integrate the section “2.1. Status quo of ESG rating system” into the introduction section and make it more concise. The current structure reduces the flow of logic and puts too much efforts in the background rather than research methods and findings.
This is a great suggestion, we have made adjustments to both the introduction and the summary,Deleted and supplemented corresponding content

2-More background should be provided regarding to the evolutionary game model. For example, why used this model and not the other models to do the analysis should be included in the introduction section.
This is a great suggestion, we have added this section.

2.3. The Development of Evolutionary Game Theory and Model Application Cases

Evolutionary game theory refers to the theory that uses mathematical knowledge to analyze the behavioral choices of game participants. In 1994, Feng Neumann and Morgenstein jointly wrote "Game Theory and Economic Behavior", constructing the theoretical system and structural framework of game theory, marking the formal establishment of game theory as a discipline. Subsequently, traditional game theory rapidly developed and was applied to various fields such as society and economy. Traditional game theory has two basic assumptions: fully rational economic agents and shared knowledge. Among them, the former refers to the behavior choices made by the game subject in order to maximize their own interests, and the game subject is an individual who almost never makes systematic wrong choices and has complete rationality. The latter refers to each game participant not only being completely rational themselves, but also considering other game participants to be completely rational. The basic assumptions of traditional game theory are almost impossible to achieve in reality, so the guiding significance of traditional game theory for many practical problems still needs to be discussed [41]. The ideological basis of evolutionary game theory is Darwin's theory of evolution. This game theory abandons the assumption of "complete rationality" in traditional game theory and believes that due to the limited cognitive ability of individuals, the game subjects are "bounded rationality". Therefore, evolutionary game theory holds that game participants cannot make behavioral choices that maximize their own interests in one game, but can continuously adjust their strategic choices through multiple repeated games, and obtain the final evolutionary stable strategy in the process of evolution. The core of evolutionary game models is to replicate dynamic equations and evolutionarily stable strategies (ESS). Copying dynamics refers to the growth rate of the proportion of people who select a certain strategy being equal to the difference between the payment obtained by that strategy and the average payment [42]. By constructing replicated dynamic equations, the evolutionary trajectory of an evolutionary game system to reach a stable equilibrium state can be derived. Evolutionary Stability Strategy (ESS) is an equilibrium strategy point that maintains a stable state. In game systems, evolutionary stable strategies are always in a stable state. This means that even if some individuals in the group experience occasional deviations, the strategy will still be in equilibrium after replicating dynamics. This paper uses an evolutionary game model to analyze the evolutionary stability strategies and paths of stakeholder games in forestry carbon sequestration, and then dissects the specific influencing factors of the construction of forestry carbon sequestration benefit sharing entities. The Stanford University Research Institute first defined the concept of "stakeholders" in 1963, stating that stakeholders are the group of people outside of the enterprise who can profit from and influence the operation of the enterprise. The research of American economist Freeman has expanded the connotation of stakeholders. In 1984, Freeman's book "Strategic Management: A Stakeholder Approach" constructed a systematic framework for stakeholder theory, marking the transformation of "stakeholders" from a concept to a comprehensive theoretical system. Freeman believes that stakeholders refer to any individual or group who can influence the achievement of organizational goals and whose behavior is also influenced by organizational goals. At this point, individuals or groups such as communities and governments, in addition to enterprises, have also been included in the research scope of stakeholder theory [43-45]. Government agencies are the makers of policies related to forestry carbon sinks, and their behavioral decisions to a certain extent affect the demand for forestry carbon sinks in society and the realization of economic benefits of forestry carbon sinks, thereby affecting the stability of effective supply of forestry carbon sinks. As the direct implementers of forestry carbon sequestration projects, the management decisions of forestry operators directly affect the effective supply of forestry carbon sequestration, thereby affecting the realization of ecological and social benefits of forestry carbon sequestration. The degree of realization of ecological, social, and economic benefits of forestry carbon sinks will also affect the willingness of forestry operators to provide forestry carbon sinks and the enthusiasm of the government to support the development of forestry carbon sinks. The emergence of independent third-party carbon sink measurement institutions is conducive to ensuring the "commodification" of carbon sinks, promoting the development of market-oriented trading of forestry carbon sinks, and ultimately facilitating the realization of ecological, social, and economic benefits of forestry carbon sinks. When the effective supply of forestry carbon sinks is insufficient or its effective supply lacks stability, third-party carbon sink measurement institutions lack development driving force and are difficult to achieve long-term development. The emergence and development of third-party carbon sink measurement institutions depend to some extent on the behavioral choices of the government and forestry operators. Therefore, in this paper, forestry carbon sink stakeholders refer to government agencies and forestry operators who have the most significant interest relationship with forestry carbon sinks.(Figure 2)

Figure 2. Logical relationship diagram of evolutionary game model.

3 Is it proper to assume and conclude the positive impact of ESG scores on the number of forestry carbon sinks purchased by enterprises? I agree with the point that a higher score will result in higher purchases. But the purchase amount itself will also increase the score. The “positive relationship” might be more accurate. Or change “amount of forestry carbon sinks purchased by enterprises” to “the potential forestry carbon sink acquisitions by enterprises”.
Under the baseline scenario, the baseline carbon layer change can be regarded as 0. Why?

This is a great suggestion, we have added a reverse example to verify:5.3 Model validation, and also replaced the expression according to your suggestion. The reviewer was very professional and rigorous,

4 Lack of references. For example, this research proposed a new application of the model. But the references for the model and its relevant parameters were not listed.
 We have made corresponding modifications and supplements

5. The article needs to be critically re-proofread. A few mistakes existed.

e.g.,

1) Titles of section 4.1.1 and 4.1.2 are the same.

2) Table 3: there is an “_” in front of the header.

3) Line 510: “andModel”.

4) Please double check the order of first and last names for all authors. I noticed there are some publications with different orders.

Thank you to the reviewer for their diligent and careful work. We have made corresponding modifications and supplements,We have made corresponding modifications and supplements

# For reviewer 3:

## First of all, thank you very much for your and the other six reviewers' suggestions. These suggestions have benefited us a lot and brought a lot of quality improvement to the article. In response to your question, I have made the following changes

1. The introduction provides a good overview of the research context, but it could benefit from a more explicit statement of the research objectives and hypotheses. How do the authors expect the incorporation of forestry carbon sequestration indicators into the ESG rating system to specifically impact enterprise behavior?

Thank you very much for the valuable comments from the reviewer. We have made corresponding modifications to the introduction, rewritten the content of this section, and supplemented it(Page3-4)

2. In the literature review section, the authors mention several studies on ESG rating systems and their impact on corporate behavior. Could they provide a more critical analysis of these studies, highlighting any gaps or contradictions in the existing literature that this research aims to address?

Thank you very much for the valuable comments from the reviewer. We have made corresponding modifications to the introduction, rewritten the content of this section, and supplemented it(Page6)

In conclusion, the existing body of research concerning the role of multiple agents in the operational mechanisms of Environmental, Social, and Governance (ESG) function realization is notably extensive, offering substantial literature support for this study. However, certain limitations persist: from a research perspective, current literature predominantly emphasizes the evaluation of the direct outcomes and advantages of ESG practices, while there is a lack of comprehensive analysis regarding the interaction mechanisms among various stakeholders. Additionally, in terms of research methodology, there is insufficient exploration into strategies for enhancing ESG practices, particularly in motivating and guiding emission control enterprises to elevate their ESG performance. Most studies tend to concentrate on macro-level policy analysis, neglecting a thorough examination of micro-level corporate decision-making behaviors and their underlying mechanisms. Furthermore, there is a scarcity of research that systematically integrates ESG practices with carbon market mechanisms to evaluate their collective impact on achieving carbon neutrality. Consequently, it remains imperative to investigate how to further activate the ESG function to effectively contribute to the realization of the "double carbon" goal. Additionally, exploring ways to stimulate market demand for forestry carbon sequestration and promote afforestation through market mechanisms and policy initiatives is essential for attaining carbon neutrality. These issues warrant in-depth study and discussion. This paper aims to construct a coupling mechanism between forestry carbon sequestration and the ESG system, utilizing an evolutionary game model for simulation to assess the mechanism's effectiveness, thereby providing a theoretical foundation and practical guidance for enhancing the ESG rating system of emission control enterprises in Guizhou and facilitating the green transformation and sustainable development of high energy-consuming industries.

3. The methodology section describes the use of the Analytic Hierarchy Process (AHP) for determining index weights. Could the authors provide more details on how the expert group for the Delphi method was selected and what criteria were used to ensure a diverse and representative panel of experts?

Thank you very much for the valuable comments from the reviewer. We have made corresponding modifications to the introduction, rewritten the content of this section, and supplemented it(Page13-14)

Perform consistency checks. Afterwards, a consistency test will be conducted, and the consistency indicators are shown in equations (3) and (4).

CI= (λmax -n)/ (n-1) (3

CR= CI/RI (4)

In equations (3) and (4), CI is the consistency index used to evaluate the consistency of the judgment matrix; CR is a random consistency ratio introduced considering the influence of n, where RI is the average random consistency index with a fixed value. If the consistency of the judgment matrix is better, the value of CR will be smaller; Usually, when CR ≤ 0.10, it means that the above judgment matrix has passed the consistency test; If CR>0.10, it indicates that the above judgment matrix has not passed the consistency test and does not have consistency. At this time, it is necessary to adjust the judgment matrix appropriately and conduct analysis and verification again. The RI standard values are 0, 0.49, 0.84, 1.15, 1.25, 1.34, 1.41, 1.45, 1.49. Since all constructed judgment matrices have passed the consistency test, the relative weight vector W of each evaluation index in the judgment matrix can be used as the corresponding weight of each evaluation index. And by calculating the weight of the lower level evaluation indicators on the overall evaluation objective based on the weight of the upper level evaluation indicators, establish an ESG rating system based on the AHP method

4. The paper mentions that "Based on the ESG reports of 400 listed companies in high energy-consuming industries" were used to construct the ESG rating system. How were these companies selected? Were there any specific criteria for inclusion or exclusion? This information would help readers understand the representativeness of the sample.

We did not specifically select these companies, we only collected large companies with high market value because they have the strength to purchase carbon credits and sufficient funds
5. In Table 3, the authors present the evaluation index of the environmental dimension under the ESG rating system. Could they elaborate on why these specific indicators were chosen and how they compare to international ESG standards? This would help readers understand the rationale behind the localized ESG system.

In the selection of ESG rating system indicators for emission control enterprises, this article collected a large amount of basic data from three dimensions: research institutions, emission control enterprises, and government units, based on the characteristics of ESG and carbon market research fields. It also invited a total of 20 experts from carbon asset management institutions, natural resource management administrative institutions, energy economy research institutions, energy conservation and emission reduction monitoring institutions, and different units of major universities to participate in the exchange and score.
6. The simulation data generation process is briefly mentioned but not fully explained. Could the authors provide more details on how the simulation data was generated and validated to ensure its reliability and representativeness of real-world scenarios?
7. In the regression analysis, the authors find that ESG scores, government subsidies, and carbon sink costs significantly impact forestry carbon sequestration purchases. Have they considered potential interaction effects between these variables? For example, does the impact of ESG scores vary depending on the level of government subsidies?
8. The evolutionary game model is a key component of this research. Could the authors provide a more detailed explanation of why this specific model was chosen over other potential methods for analyzing the interaction between enterprises, governments, and investment institutions?
9. In the results section, the authors state that "The stable points of the evolutionary game are E5 (1,1,0) and E6 (1,0,1)." Could they provide a more detailed interpretation of what these stable points mean in practical terms for the behavior of enterprises, governments, and investment institutions?
10. The paper discusses heterogeneity in different industries regarding the impact of various factors on forestry carbon sequestration purchases. Have the authors considered conducting a more in-depth analysis of why these differences exist and what implications they might have for industry-specific policies?

This is a great combination of suggested questions, and we have made corresponding modifications in the article. At the same time, we have added a reverse evolution case to demonstrate the effectiveness and consistency of our evolution

5.3. Model validation

The model used in this article belongs to the classic model in evolutionary game theory and has been extensively used by researchers[46-51].To further explore this issue, this paper utilized Chen's data and conducted numerical simulation analysis. MATLAB was used to perform the Pareto optimal state combination mentioned above to verify the effectiveness of evolutionary game stability analysis and the sensitivity of each agent to parameters.[52]

We shift our focus to the buyers of forestry carbon sinks:Coal Power Enterprises  (CPEs) and New Energy Power Enterprises(NEPEs).According to Chen's calculation simulation:Under the condition of satisfying the parameter setting conditions of Pareto opti mality, this article considers the actual situation and relevant expert opinions compre hensively, and the initial values of the parameters are as follows: R1= 2, R2= 4, Rs= 3, C1= 2, C2= 1, Cg= 4, A1= 3, A2= 2, B1= 3, B2= 3, F1= 2, F2= 3. The above parameters are brought into the tripartite game evolution system, and numerical simulation analysis is conducted with MATLAB R2021a software. The results are shown in Figure 3.

Figure 3. Parameter evolution simulation results.

[Figure 3](https://www.mdpi.com/1996-1073/17/18/4553#fig_body_display_energies-17-04553-f002) illustrates the evolutionary trend in the tripartite game under joint operation, where it can be seen that the initial NEPE had a stronger inclination to choose joint operation due to the low-cost supervision value of the CPE. The government initially preferred a loose supervision strategy, and as the other two entities’ willingness to choose joint operation increased, the government’s willingness to choose a strict supervision strategy is also increased. When the NEPE evolved to joint operation, the government’s rate of choosing strict supervision was faster than that of CPE choosing joint operation. [Figure 2](https://www.mdpi.com/1996-1073/17/18/4553#fig_body_display_energies-17-04553-f002) shows that the system ultimately tends towards the ideal state of (1, 1, 1), where the CPE chooses joint operation, the NEPE chooses joint operation, and the government implements strict supervision, indicating the validity of the conclusion.

[Figure 4](https://www.mdpi.com/1996-1073/17/18/4553#fig_body_display_energies-17-04553-f003) shows that with the increase in additional operating income that the CPE can obtain through joint operation, its willingness to choose joint operation becomes stronger, and government is more willing to choose strict supervision.

[Figure 5](https://www.mdpi.com/1996-1073/17/18/4553#fig_body_display_energies-17-04553-f004) shows that with the increase in additional operating income that the NEPE can obtain through joint operation, its willingness to choose joint operation becomes stronger, and the government has a greater willingness to choose strict supervision.These simulation conclusions are consistent with our model calculation conclusions, proving the effectiveness and reliability of the model

Figure 4. The influence of the additional operating income of the CPE choosing joint operation.

Figure 5. The influence of the additional operating income of the NEPE choosing joint operation.

11. In the conclusion, the authors state that "the ESG rating system after the introduction of forestry carbon sequestration can spontaneously promote the purchase of forestry carbon sequestration by emission control enterprises from the market mechanism." Could they provide more evidence or explanation to support this claim?
12. The policy implications section provides several recommendations. Could the authors discuss potential challenges or barriers to implementing these recommendations, particularly in the context of China's current regulatory environment?

We have made modifications to the above two issues in the conclusion section of the article
13. The study could be improved by integrating insights from recent research in related fields: <https://doi.org/10.3390/ma15207098;> <https://doi.org/10.1016/j.conbuildmat.2023.132604;> <https://doi.org/10.1016/j.clema.2022.100111>

Very good article, I have learned a lot from it and it is very useful for discussing the conclusions of the article. I have cited them in references [53-56]
14. The study focuses on the environmental dimension of ESG ratings. Have the authors considered how the social and governance dimensions might interact with or influence the environmental aspects, particularly in relation to forestry carbon sequestration?
15. While the paper provides valuable insights, it would benefit from a more explicit discussion of its limitations and potential areas for future research. For example, how might the findings be affected by changes in carbon pricing or technological advancements in carbon capture?

Great suggestion, we did not cover these two directions in this study. We will consider conducting research in these two directions in the future. Thank you again for your valuable suggestion

# For reviewer 4:

First of all, thank you very much for your and the other six reviewers' suggestions. These suggestions have benefited us a lot and brought a lot of quality improvement to the article. From the review comments, Reviewer 4 was very serious and professional, providing many constructive and excellent suggestions. I have supplemented and revised the content of the article according to his suggestions. Thank you very much

Abstract
1. Page 1, Lines 12-26:
o Clarity: The abstract is somewhat dense and could benefit from clearer language. For instance, the sentence, "the ESG score and the amount of government subsidies have a significant positive impact on the amount of forestry carbon sequestration purchased by enterprises..." could be simplified for a broader audience.
o Recommendation: Consider breaking down complex ideas into simpler statements and possibly reducing jargon.
Introduction

Corresponding modifications have been made, with some content deleted and rewritten and supplemented according to the reviewer's comments. For details, please refer to the article's changes and revisions
2. Page 2, Lines 33-91:
o Context: The introduction effectively outlines the importance of China's "double carbon" goals and sets the stage for the research. However, there is a lack of international context. How do China's efforts compare to other countries?
o Recommendation: Briefly mention global efforts in forestry carbon sequestration to provide international context.

Corresponding modifications have been made, with some content deleted and rewritten and supplemented according to the reviewer's comments. For details, please refer to the article's changes and revisions

3. Page 2, Line 86-91:
o Linkage: While the introduction covers the macro and micro aspects of carbon sequestration and ESG, the linkage between these two levels can be made clearer.
o Recommendation: Explicitly outline how the macro and micro designs combine to create the proposed mechanism.

Corresponding modifications have been made, with some content deleted and rewritten and supplemented according to the reviewer's comments. For details, please refer to the article's changes and revisions

Literature Review
4. Page 3-4, Lines 92-205:
o Critical Review: The literature review is comprehensive, but it could benefit from more critical analysis of the gaps in previous research, particularly regarding the shortcomings of ESG systems globally.
o Recommendation: Strengthen the critique of past research to better justify the need for the current study.
Methodology

Corresponding modifications have been made, with some content deleted and rewritten and supplemented according to the reviewer's comments. For details, please refer to the article's changes and revisions

5. Page 6, Lines 207-248:
o Game Model Clarification: The operation mechanism and evolutionary game model are central to the paper, but the explanation is overly complex and technical. Simplification and additional explanation would benefit readers unfamiliar with game theory.
o Recommendation: Provide a short primer on evolutionary game theory to help readers who may not be familiar with it. Adding a diagram could also aid understanding.

Corresponding modifications have been made, with some content deleted and rewritten and supplemented according to the reviewer's comments. For details, please refer to the article's changes and revisions
2.3. The Development of Evolutionary Game Theory and Model Application Cases

Evolutionary game theory refers to the theory that uses mathematical knowledge to analyze the behavioral choices of game participants. In 1994, Feng Neumann and Morgenstein jointly wrote "Game Theory and Economic Behavior", constructing the theoretical system and structural framework of game theory, marking the formal establishment of game theory as a discipline. Subsequently, traditional game theory rapidly developed and was applied to various fields such as society and economy. Traditional game theory has two basic assumptions: fully rational economic agents and shared knowledge. Among them, the former refers to the behavior choices made by the game subject in order to maximize their own interests, and the game subject is an individual who almost never makes systematic wrong choices and has complete rationality. The latter refers to each game participant not only being completely rational themselves, but also considering other game participants to be completely rational. The basic assumptions of traditional game theory are almost impossible to achieve in reality, so the guiding significance of traditional game theory for many practical problems still needs to be discussed [41]. The ideological basis of evolutionary game theory is Darwin's theory of evolution. This game theory abandons the assumption of "complete rationality" in traditional game theory and believes that due to the limited cognitive ability of individuals, the game subjects are "bounded rationality". Therefore, evolutionary game theory holds that game participants cannot make behavioral choices that maximize their own interests in one game, but can continuously adjust their strategic choices through multiple repeated games, and obtain the final evolutionary stable strategy in the process of evolution. The core of evolutionary game models is to replicate dynamic equations and evolutionarily stable strategies (ESS). Copying dynamics refers to the growth rate of the proportion of people who select a certain strategy being equal to the difference between the payment obtained by that strategy and the average payment [42]. By constructing replicated dynamic equations, the evolutionary trajectory of an evolutionary game system to reach a stable equilibrium state can be derived. Evolutionary Stability Strategy (ESS) is an equilibrium strategy point that maintains a stable state. In game systems, evolutionary stable strategies are always in a stable state. This means that even if some individuals in the group experience occasional deviations, the strategy will still be in equilibrium after replicating dynamics. This paper uses an evolutionary game model to analyze the evolutionary stability strategies and paths of stakeholder games in forestry carbon sequestration, and then dissects the specific influencing factors of the construction of forestry carbon sequestration benefit sharing entities. The Stanford University Research Institute first defined the concept of "stakeholders" in 1963, stating that stakeholders are the group of people outside of the enterprise who can profit from and influence the operation of the enterprise. The research of American economist Freeman has expanded the connotation of stakeholders. In 1984, Freeman's book "Strategic Management: A Stakeholder Approach" constructed a systematic framework for stakeholder theory, marking the transformation of "stakeholders" from a concept to a comprehensive theoretical system. Freeman believes that stakeholders refer to any individual or group who can influence the achievement of organizational goals and whose behavior is also influenced by organizational goals. At this point, individuals or groups such as communities and governments, in addition to enterprises, have also been included in the research scope of stakeholder theory [43-45]. Government agencies are the makers of policies related to forestry carbon sinks, and their behavioral decisions to a certain extent affect the demand for forestry carbon sinks in society and the realization of economic benefits of forestry carbon sinks, thereby affecting the stability of effective supply of forestry carbon sinks. As the direct implementers of forestry carbon sequestration projects, the management decisions of forestry operators directly affect the effective supply of forestry carbon sequestration, thereby affecting the realization of ecological and social benefits of forestry carbon sequestration. The degree of realization of ecological, social, and economic benefits of forestry carbon sinks will also affect the willingness of forestry operators to provide forestry carbon sinks and the enthusiasm of the government to support the development of forestry carbon sinks. The emergence of independent third-party carbon sink measurement institutions is conducive to ensuring the "commodification" of carbon sinks, promoting the development of market-oriented trading of forestry carbon sinks, and ultimately facilitating the realization of ecological, social, and economic benefits of forestry carbon sinks. When the effective supply of forestry carbon sinks is insufficient or its effective supply lacks stability, third-party carbon sink measurement institutions lack development driving force and are difficult to achieve long-term development. The emergence and development of third-party carbon sink measurement institutions depend to some extent on the behavioral choices of the government and forestry operators. Therefore, in this paper, forestry carbon sink stakeholders refer to government agencies and forestry operators who have the most significant interest relationship with forestry carbon sinks.(Figure 2)

Figure 2. Logical relationship diagram of evolutionary game model.

6. Page 9, Line 407:
o Weighting Criteria: The paper uses the Analytic Hierarchy Process (AHP) to determine weights in the ESG rating system. The AHP process is mentioned, but further details on how experts were chosen and how scores were aggregated are needed.
o Recommendation: Provide more detail on the AHP process, including how experts were selected and how subjectivity was minimized.

In the selection of ESG rating system indicators for emission control enterprises, this article collected a large amount of basic data from three dimensions: research institutions, emission control enterprises, and government units, based on the characteristics of ESG and carbon market research fields. It also invited a total of 20 experts from carbon asset management institutions, natural resource management administrative institutions, energy economy research institutions, energy conservation and emission reduction monitoring institutions, and different units of major universities to participate in the exchange and score.

Results
7. Page 14, Lines 572-602:
o Descriptive Statistics: The results section is clear, but the descriptive statistics could benefit from more discussion. How do these statistics compare with existing data or studies in this field?
o Recommendation: Compare the descriptive statistics with similar studies to highlight the uniqueness of your findings.
Discussion
8. Page 15, Lines 626-655:
o Policy Implications: The discussion mentions policy implications, but they are somewhat general.
o Recommendation: Be more specific about the implications of your findings for policymakers, particularly in terms of forestry carbon sequestration policies in China and abroad.
9. Page 15-16, Lines 633-641:
o Industry Comparison: The heterogeneity analysis across industries is a strong point of the paper. However, more detail is needed on why certain industries (e.g., chemical or textile industries) are affected differently.
o Recommendation: Expand on the reasons behind the varying effects of ESG and government subsidies across different industries.

We have revised our conclusions based on these suggestions and issues, and added a chapter before the discussion to demonstrate the reliability and effectiveness through inversion
Writing and Organization
10. General Writing:
o Language: There are several instances where the language is overly technical or complex.
o Recommendation: Simplify language and sentence structure throughout the manuscript for broader accessibility.
11. Structure: The manuscript is well-structured but would benefit from clearer section transitions.
o Recommendation: Use more explicit headings or sub-headings to break up dense sections, particularly in the methodology and results.
In general, the study provides important insights into forestry carbon sequestration in China and offers a novel approach using an evolutionary game model. However, improvements can be made in terms of clarity, depth of analysis, and the presentation of results.

Thank you again for your work. You are a passionate and dedicated scholar,We have optimized the article and have also contacted the official proofreading company for professional correction and optimization before publication

# For reviewer 5:

## First of all, thank you very much for your and the other six reviewers' suggestions. These suggestions have benefited us a lot and brought a lot of quality improvement to the article. In response to your question, I have made the following changes

1. Abbreviations are used in the abstract or at most places of the paper. Interpret for the first time and then use abbreviations e.g. ESG, CCER and etc.

Corresponding modifications have been made, with some content deleted and rewritten and supplemented according to the reviewer's comments. For details, please refer to the article's changes and revisions
ESG:Environmental, Social and Governance

CCER :China Certified Emission Reduction
2. In the abstract section, the need and scope of the study should be included. At the end of the abstract include some quantitative results

Revised this section and presented novelty and methodology

Guizhou Province has abundant forest resources, and it has great economic value and social benefits to explore the practical path of forestry carbon sequestration. Based on the current situation of forestry carbon sequestration development in Guizhou Province, this paper innovatively integrates forestry carbon sequestration indicators into the existing Environmental, Social and Governance(ESG) evaluation system using an evolutionary game model. It analyzes the factors restricting forestry carbon sequestration and explores the influencing factors of forestry carbon sequestration benefit sharing bodies in Guizhou. Through regression analysis, the paper discusses the impact of enterprise ESG scores, government subsidy amounts, and forestry carbon sequestration costs on forestry carbon sequestration purchase volume. The research results show that enterprise ESG scores and government subsidy amounts have a significant positive impact on enterprise forestry carbon sequestration purchase volume, while forestry carbon sequestration costs have a significant negative impact. The results have passed the robustness test in different industries. The simulation analysis results show that the stable point of the evolutionary game is (1,0,1) and (1,1,0), which verifies that the ESG rating system with forestry carbon sequestration integration can promote enterprises to purchase more forestry carbon sequestration, i.e., the effectiveness of forestry carbon sequestration in activating the ESG rating system mechanism. Based on the research conclusions, the paper puts forward policy implications: the government should accelerate the construction of localized ESG rating systems, improve enterprise information disclosure and supervision, increase subsidies and reduce forestry carbon sequestration costs, and optimize carbon quota design.
3. Check the Keywords. What do you mean by Keywords ESG.

Corresponding modifications have been made
4. The objectives and research insight questions must be stated at the last paragraph of introduction section.

Rewrite this part of the content(Page2-3)

5. What are specific values in the evolutionary game parameter assumptions and what is the criteria to choose values of these parameters.

These are mostly empirical parameters and existing values. The specific values calculated this time are the carbon emissions of high carbon enterprises
6. How are the specified values in Table 3 obtained? Do these values have significant physical meaning?

In the selection of ESG rating system indicators for emission control enterprises, this article collected a large amount of basic data from three dimensions: research institutions, emission control enterprises, and government units, based on the characteristics of ESG and carbon market research fields. It also invited a total of 20 experts from carbon asset management institutions, natural resource management administrative institutions, energy economy research institutions, energy conservation and emission reduction monitoring institutions, and different units of major universities to participate in the exchange and score.

# For reviewer 6:

First of all, thank you very much for your and the other six reviewers' suggestions. These suggestions have benefited us a lot and brought a lot of quality improvement to the article. From the review comments, Reviewer 6 was very serious and professional, providing many constructive and excellent suggestions. I have supplemented and revised the content of the article according to his suggestions. Thank you very much

Suggestions for Improvement:
1. While the manuscript mentions field surveys and interviews, more details can be provided on the data collection process, sample characteristics, questionnaire design, etc. to enhance the credibility of the qualitative inputs used in the evolutionary game parameter setting.
This is a great suggestion, but we collected too much data in the early stages and didn't list them all. If there are readers who need it later, we will provide it
2. The description of the simulation data generation process for testing influencing factors can be further elaborated. Providing the codes/equations used to generate the simulation data in an appendix would allow replicability.
3. Perform sensitivity analysis on key parameters of the evolutionary game model to test the robustness of the findings. Discuss how changes in parameters like government subsidies, carbon sequestration costs, etc. impact the equilibrium outcomes.
4. Discuss the limitations of the study more explicitly. For example, the evolutionary game assumes rational behavior but bounded rationality of actors can influence outcomes. The simulation is a simplified representation of the real dynamics. Highlighting such limitations helps interpret the findings appropriately.
5. Proofread the manuscript thoroughly to fix minor language and grammatical errors. Ensure consistency in abbreviations, figure and table numbers, references, etc.

These four suggestions are all very good. Based on these suggestions, we have made significant changes to our article, adding nearly 10 pages of content and deleting unnecessary expressions and explanations,I have highlighted the modified content in red in the text

Title and Abstract:Rewrite this part of the content

Introduction (Section 1):Rewrite this part of the content

Theoretical Analysis Framework and Research Methods:Corresponding modifications have been made

Analysis and Simulation (Section 5):

- The benchmark regression results (Table 6, lines 589-626) support the research hypotheses. However, the model specification is not provided. Specify the regression equation and discuss the choice of control variables, if any.
- The heterogeneity analysis (Table 7, lines 627-657) is a useful extension but lacks depth. Discuss the industry-specific findings in more detail and relate them to the characteristics of each industry (limit it to 1 or 2 paragraphs).

This is a great combination of suggested questions, and we have made corresponding modifications in the article. At the same time, we have added a reverse evolution case to demonstrate the effectiveness and consistency of our evolution

“Consider the following articles which could help strengthen your research and literature review”
• <https://doi.org/10.1016/j.egyr.2024.08.031>
• <https://doi.org/10.1016/j.cacint.2023.100127>
Very good article, I have learned a lot from it and it is very useful for discussing the conclusions of the article. I have cited them in references [57-58]

# For reviewer 7:

## First of all, thank you very much for your and the other six reviewers' suggestions. These suggestions have benefited us a lot and brought a lot of quality improvement to the article. In response to your question, I have made the following changes

1. However, there is some lack of clarity in data presentation. E.g. the manuscript does not provide sufficient clarity on the source and handling of the data, especially regarding parameter assumptions in the game model, which may affect the robustness of the results.

This is a good question about the validity and consistency of the data. To verify this issue, we have added an inversion chapter, and the conclusions are relatively consistent

The model used in this article belongs to the classic model in evolutionary game theory and has been extensively used by researchers[46-51].To further explore this issue, this paper utilized Chen's data and conducted numerical simulation analysis. MATLAB was used to perform the Pareto optimal state combination mentioned above to verify the effectiveness of evolutionary game stability analysis and the sensitivity of each agent to parameters.[52]

We shift our focus to the buyers of forestry carbon sinks:Coal Power Enterprises  (CPEs) and New Energy Power Enterprises(NEPEs).According to Chen's calculation simulation:Under the condition of satisfying the parameter setting conditions of Pareto opti mality, this article considers the actual situation and relevant expert opinions compre hensively, and the initial values of the parameters are as follows: R1= 2, R2= 4, Rs= 3, C1= 2, C2= 1, Cg= 4, A1= 3, A2= 2, B1= 3, B2= 3, F1= 2, F2= 3. The above parameters are brought into the tripartite game evolution system, and numerical simulation analysis is conducted with MATLAB R2021a software. The results are shown in Figure 3.

Figure 3. Parameter evolution simulation results.

[Figure 3](https://www.mdpi.com/1996-1073/17/18/4553#fig_body_display_energies-17-04553-f002) illustrates the evolutionary trend in the tripartite game under joint operation, where it can be seen that the initial NEPE had a stronger inclination to choose joint operation due to the low-cost supervision value of the CPE. The government initially preferred a loose supervision strategy, and as the other two entities’ willingness to choose joint operation increased, the government’s willingness to choose a strict supervision strategy is also increased. When the NEPE evolved to joint operation, the government’s rate of choosing strict supervision was faster than that of CPE choosing joint operation. [Figure 2](https://www.mdpi.com/1996-1073/17/18/4553#fig_body_display_energies-17-04553-f002) shows that the system ultimately tends towards the ideal state of (1, 1, 1), where the CPE chooses joint operation, the NEPE chooses joint operation, and the government implements strict supervision, indicating the validity of the conclusion.

[Figure 4](https://www.mdpi.com/1996-1073/17/18/4553#fig_body_display_energies-17-04553-f003) shows that with the increase in additional operating income that the CPE can obtain through joint operation, its willingness to choose joint operation becomes stronger, and government is more willing to choose strict supervision.

[Figure 5](https://www.mdpi.com/1996-1073/17/18/4553#fig_body_display_energies-17-04553-f004) shows that with the increase in additional operating income that the NEPE can obtain through joint operation, its willingness to choose joint operation becomes stronger, and the government has a greater willingness to choose strict supervision.These simulation conclusions are consistent with our model calculation conclusions, proving the effectiveness and reliability of the model

Figure 4. The influence of the additional operating income of the CPE choosing joint operation.

Figure 5. The influence of the additional operating income of the NEPE choosing joint operation.

2. The study is geographically limited to Guizhou, which may reduce the generalizability of the findings to other regions with different regulatory or economic environments. There should be some scenarios with different conditions in discussion part of the manuscript.

The policy has strong regional specificity and requires the foundation of ecological civilization construction and the support of national policies. At the same time, it requires enterprises and finance to have certain policies. The scope of adaptation is relatively limited and not suitable for promotion. It can only be one place, one policy measure, and one solution
3. While the discussion of policy implications is strong, the explanation of certain technical details in the game model and the results is lacking in depth, which could confuse readers unfamiliar with such models.

Added a chapter to explain this issue：2.3. The Development of Evolutionary Game Theory and Model Application Cases

Evolutionary game theory refers to the theory that uses mathematical knowledge to analyze the behavioral choices of game participants. In 1994, Feng Neumann and Morgenstein jointly wrote "Game Theory and Economic Behavior", constructing the theoretical system and structural framework of game theory, marking the formal establishment of game theory as a discipline. Subsequently, traditional game theory rapidly developed and was applied to various fields such as society and economy. Traditional game theory has two basic assumptions: fully rational economic agents and shared knowledge. Among them, the former refers to the behavior choices made by the game subject in order to maximize their own interests, and the game subject is an individual who almost never makes systematic wrong choices and has complete rationality. The latter refers to each game participant not only being completely rational themselves, but also considering other game participants to be completely rational. The basic assumptions of traditional game theory are almost impossible to achieve in reality, so the guiding significance of traditional game theory for many practical problems still needs to be discussed [41]. The ideological basis of evolutionary game theory is Darwin's theory of evolution. This game theory abandons the assumption of "complete rationality" in traditional game theory and believes that due to the limited cognitive ability of individuals, the game subjects are "bounded rationality". Therefore, evolutionary game theory holds that game participants cannot make behavioral choices that maximize their own interests in one game, but can continuously adjust their strategic choices through multiple repeated games, and obtain the final evolutionary stable strategy in the process of evolution. The core of evolutionary game models is to replicate dynamic equations and evolutionarily stable strategies (ESS). Copying dynamics refers to the growth rate of the proportion of people who select a certain strategy being equal to the difference between the payment obtained by that strategy and the average payment [42]. By constructing replicated dynamic equations, the evolutionary trajectory of an evolutionary game system to reach a stable equilibrium state can be derived. Evolutionary Stability Strategy (ESS) is an equilibrium strategy point that maintains a stable state. In game systems, evolutionary stable strategies are always in a stable state. This means that even if some individuals in the group experience occasional deviations, the strategy will still be in equilibrium after replicating dynamics. This paper uses an evolutionary game model to analyze the evolutionary stability strategies and paths of stakeholder games in forestry carbon sequestration, and then dissects the specific influencing factors of the construction of forestry carbon sequestration benefit sharing entities. The Stanford University Research Institute first defined the concept of "stakeholders" in 1963, stating that stakeholders are the group of people outside of the enterprise who can profit from and influence the operation of the enterprise. The research of American economist Freeman has expanded the connotation of stakeholders. In 1984, Freeman's book "Strategic Management: A Stakeholder Approach" constructed a systematic framework for stakeholder theory, marking the transformation of "stakeholders" from a concept to a comprehensive theoretical system. Freeman believes that stakeholders refer to any individual or group who can influence the achievement of organizational goals and whose behavior is also influenced by organizational goals. At this point, individuals or groups such as communities and governments, in addition to enterprises, have also been included in the research scope of stakeholder theory [43-45]. Government agencies are the makers of policies related to forestry carbon sinks, and their behavioral decisions to a certain extent affect the demand for forestry carbon sinks in society and the realization of economic benefits of forestry carbon sinks, thereby affecting the stability of effective supply of forestry carbon sinks. As the direct implementers of forestry carbon sequestration projects, the management decisions of forestry operators directly affect the effective supply of forestry carbon sequestration, thereby affecting the realization of ecological and social benefits of forestry carbon sequestration. The degree of realization of ecological, social, and economic benefits of forestry carbon sinks will also affect the willingness of forestry operators to provide forestry carbon sinks and the enthusiasm of the government to support the development of forestry carbon sinks. The emergence of independent third-party carbon sink measurement institutions is conducive to ensuring the "commodification" of carbon sinks, promoting the development of market-oriented trading of forestry carbon sinks, and ultimately facilitating the realization of ecological, social, and economic benefits of forestry carbon sinks. When the effective supply of forestry carbon sinks is insufficient or its effective supply lacks stability, third-party carbon sink measurement institutions lack development driving force and are difficult to achieve long-term development. The emergence and development of third-party carbon sink measurement institutions depend to some extent on the behavioral choices of the government and forestry operators. Therefore, in this paper, forestry carbon sink stakeholders refer to government agencies and forestry operators who have the most significant interest relationship with forestry carbon sinks.(Figure 2)

Figure 2. Logical relationship diagram of evolutionary game model.

4. The manuscript has potential, but significant revisions are needed to clarify data sources, improve the technical explanation of the model, and broaden the discussion to make the findings applicable beyond Guizhou Province.

Thank you very much for the reviewer's suggestions. We will continue to explore in future research, which is also the research direction we want to do in the future
