# Supplementary material for: Exploration of forestry carbon sequestration practice path in Guizhou province-based on evolutionary game model
Source: PLoS One. 2024 Dec 13;19(12):e0314805. doi: 10.1371/journal.pone.0314805 (PMC11643302; doi:10.1371/journal.pone.0314805)
Supplement: Attachment — Submitted filename: Response to Reviewers11.14.doc [file pone.0314805.s004.doc]

Dear editor：

Thank you again for your valuable suggestions and guidance, as well as those of the reviewers. We have learned much from those comments, which are fair, encouraging and constructive. After carefully studying the comments and your advice, we have made corresponding changes. The main revisions are listed below.

# For reviewer 1:

Reviewer #1: All comments were addressed by the author. I recommend for accept this manuscript for publish
**Thank you for your guidance and assistance. The improvement of the quality of the article cannot be achieved without the valuable suggestions of such a professional reviewer. thank you for your work**

# For reviewer 2:

Reviewer #2: Thanks for all the responses. Only one final suggestion: the background of the evolutionary game model might be too basic and detailed. It is recommended to keep only those closely related to this research.

This is a great suggestion, Our description is a bit too basic and detailed, we have deleted the basic knowledge points in 2.3,Delete the following content:Evolutionary game theory refers to the theory that uses mathematical knowledge to analyze the behavioral choices of game participants. In 1994, Feng Neumann and Morgenstein jointly wrote "Game Theory and Economic Behavior", constructing the theoretical system and structural framework of game theory, marking the formal establishment of game theory as a discipline. Subsequently, traditional game theory rapidly developed and was applied to various fields such as society and economy. Traditional game theory has two basic assumptions: fully rational economic agents and shared knowledge. Among them, the former refers to the behavior choices made by the game subject in order to maximize their own interests, and the game subject is an individual who almost never makes systematic wrong choices and has complete rationality. The latter refers to each game participant not only being completely rational themselves, but also considering other game participants to be completely rational. The basic assumptions of traditional game theory are almost impossible to achieve in reality, so the guiding significance of traditional game theory for many practical problems still needs to be discussed [41]. The ideological basis of evolutionary game theory is Darwin's theory of evolution. This game theory abandons the assumption of "complete rationality" in traditional game theory and believes that due to the limited cognitive ability of individuals, the game subjects are "bounded rationality". Therefore, evolutionary game theory holds that game participants cannot make behavioral choices that maximize their own interests in one game, but can continuously adjust their strategic choices through multiple repeated games, and obtain the final evolutionary stable strategy in the process of evolution.

# For reviewer 4:

Reviewer #4: This manuscript is now acceptable for publication. However, in the data availability statement, the authors stated that 'All relevant data are within the manuscript and its Supporting Information files.' I could not find the data in the manuscript or in the supporting files.
Thank you very much for the valuable comments from the reviewer. Some of the data in this article was extracted from the Guizhou Provincial Forestry Yearbook. We have translated these forestry yearbooks into English and submitted them as supporting materials

# For reviewer 5:

Reviewer #5: I have thoroughly reviewed the revised manuscript, and I appreciate the substantial revisions that have been made to address all critical areas. However, I accept the revised paper for publication.

**Thank you for your guidance and assistance. The improvement of the quality of the article cannot be achieved without the valuable suggestions of such a professional reviewer. thank you for your work**

# For reviewer 6:

Reviewer #6: Re-review of the manuscript Exploration of Forestry Carbon Sequestration Practice Path in Guizhou Province Based on Evolutionary Game Model based on the comments I provided and the authors' responses. I would agree that the authors have addressed my comments and I would recommend accepting the manuscript for publication in PLOS ONE.

**Thank you for your guidance and assistance. The improvement of the quality of the article cannot be achieved without the valuable suggestions of such a professional reviewer. thank you for your work**

Thank you very much for the reviewer's suggestions. We will continue to explore in future research, which is also the research direction we want to do in the future
